# PSReliP: an integrated pipeline for analysis and visualization of population structure and relatedness based on genome-wide genetic variant data

**DOI:** 10.1186/s12859-023-05169-4

**Published:** 2023-04-05

**Authors:** Elena Solovieva, Hiroaki Sakai

**Affiliations:** Research Center for Advanced Analysis, National Agriculture and Food Research Organization, Tsukuba, Ibaraki Japan

**Keywords:** Population stratification, Cryptic relatedness, Population structure analysis, Genetic relatedness investigation, Data visualization, PLINK, Shiny application, Bioinformatics pipeline

## Abstract

**Background:**

Population structure and cryptic relatedness between individuals (samples) are two major factors affecting false positives in genome-wide association studies (GWAS). In addition, population stratification and genetic relatedness in genomic selection in animal and plant breeding can affect prediction accuracy. The methods commonly used for solving these problems are principal component analysis (to adjust for population stratification) and marker-based kinship estimates (to correct for the confounding effects of genetic relatedness). Currently, many tools and software are available that analyze genetic variation among individuals to determine population structure and genetic relationships. However, none of these tools or pipelines perform such analyses in a single workflow and visualize all the various results in a single interactive web application.

**Results:**

We developed PSReliP, a standalone, freely available pipeline for the analysis and visualization of population structure and relatedness between individuals in a user-specified genetic variant dataset. The analysis stage of PSReliP is responsible for executing all steps of data filtering and analysis and contains an ordered sequence of commands from PLINK, a whole-genome association analysis toolset, along with in-house shell scripts and Perl programs that support data pipelining. The visualization stage is provided by Shiny apps, an R-based interactive web application. In this study, we describe the characteristics and features of PSReliP and demonstrate how it can be applied to real genome-wide genetic variant data.

**Conclusions:**

The PSReliP pipeline allows users to quickly analyze genetic variants such as single nucleotide polymorphisms and small insertions or deletions at the genome level to estimate population structure and cryptic relatedness using PLINK software and to visualize the analysis results in interactive tables, plots, and charts using Shiny technology. The analysis and assessment of population stratification and genetic relatedness can aid in choosing an appropriate approach for the statistical analysis of GWAS data and predictions in genomic selection. The various outputs from PLINK can be used for further downstream analysis. The code and manual for PSReliP are available at https://github.com/solelena/PSReliP.

**Supplementary Information:**

The online version contains supplementary material available at 10.1186/s12859-023-05169-4.

## Background

### Overview of topics of PSReliP

Population structure (or population stratification) (PS) and cryptic relatedness (CR) are two basic aspects of population genetics. PS refers to the presence of systematic differences in allele frequencies between subpopulations that arise from non-random mating. CR (unknown to the investigators) occurs when some individuals are closely related, but this close relatedness is unreported. PS and CR can lead to the problem of confounding in genetic association studies [1]. A genome-wide association study (GWAS) is an approach used to evaluate the associations between specific genetic variants and particular phenotypes or diseases.

Principal components analysis (PCA) is the most widely used method to adjust for PS in GWAS [2]. In genetic studies, PCA is generally applied to a genomic relationship matrix (GRM). The method used in the PLINK 1.9 [3] and 2.0 [4] for the computation of the variance-standardized GRM is similar to the method implemented in the GCTA [5] (genome-wide complex trait analysis) tool [6]. The GRM, estimated using GCTA and PLINK software, can be interpreted as a matrix representation of genetic relationships between individuals in a specified dataset of genetic variants (https://cnsgenomics.com/software/gcta/#Overview).

Related approaches, such as multidimensional scaling (MDS) performed on identity-by-state (IBS) pairwise distances, can also be applied to control PS in GWAS [7]. Several examples of using MDS for PS analysis have been presented in scientific literature. For example, Linge et al. [8] used MDS to investigate PS using a dataset of 620 individuals from several peach cultivars. In that study, PS was analyzed using MDS and clustering analyses.

Both MDS and cluster analyses can be performed based on IBS pairwise distances (the genome-wide average proportion of alleles sharing IBS between any two individuals) [9]. IBS analysis is a widely used and easily applicable method to measure genetic similarity (similarity of alleles) between pairs of individuals in a population (IBS alleles are not necessarily a consequence of identity by descent (IBD)). This analysis may help understand the degree of genetic diversity in the whole population and different subpopulations.

The kinship coefficient, defined as the probability that two homologous alleles, one from each of two individuals, are identical by descent (IBD), is a classic measurement of relatedness (genetic relationships among individuals resulting from shared ancestry) and is important in many fields of biology [10]. Genetic relatedness can be calculated from the pedigree (the pedigree-based kinship) or can be estimated using genetic marker data (the marker-based kinship). Pedigree-free (marker-based) methods are preferred for estimating kinship coefficients when there are difficulties in restoring pedigrees in natural populations, or when the results of kinship analysis are used to infer relatedness in GWAS with unavailable or inaccurate pedigree information [1]. Several methods have been developed to estimate kinship coefficients from the genotypic data [1, 11].

Common estimation approaches use allele frequencies for kinship estimation, meaning that an appropriate reference population is required [11]. Other kinship estimation methods, such as the KING-robust estimator [12, 13], do not use allele frequencies and can provide robust relationship inference in the presence of an unknown population substructure.

Marker-based kinship coefficient matrices (a matrix that contains the pairwise kinship coefficient between all individuals) can be used to correct hidden relatedness as a random effect in a mixed-model approach for GWAS analysis [14]. The mixed-model approach, which accounts for confounding factors such as fixed effects (for PS) and random effects (kinship matrix), has been widely used in GWAS [15–17], particularly in GWAS conducted in plants and animals. In addition, PS and CR are factors that can influence the prediction of genomic selection (GS) [18–20]. Wright’s F-statistics, including Wright's fixation index (FST), is one of the most used statistics in population and evolutionary genetics [21]. F-statistics, particularly FST, is commonly used to measure genetic variation in different populations (PS or the genetic differentiation of populations) [21–23].

The coefficient of inbreeding (F) of an individual is a measure of inbreeding and can be defined as the probability that two alleles at any given locus in an individual are IBD [24–26]. Estimating the inbreeding coefficients of individuals in GWAS data is important for quality control (QC) when deciding whether to remove individuals with highly positive or highly negative inbreeding coefficients. Highly positive inbreeding coefficients indicated many homozygous genotypes and high levels of inbreeding. The inclusion of these individuals can influence GWAS results because the random mating assumption required for the standard GWAS test is violated. Highly negative inbreeding coefficients, which can be calculated by some estimators, indicate too many heterozygous genotypes and suggest the possibility of contamination.

### Integrated approach to data analysis and visualization

In population genetics research and GWAS analysis, several analytical tools and software packages have been developed to investigate the stratification and relatedness in the population genetics studies. PLINK [9, 27] is a popular and commonly used program for analyzing genetic variant data, including the detection of PS and CR. However, PLINK (like many other bioinformatics tools) provides the user with many commands to perform various analyses that require a deep understanding of the available parameters, their combinations, supported file formats, etc. To perform an in-depth computational analysis, it is necessary to execute several commands sequentially, with additional steps for data selection and filtering, changing data formats, etc.

In addition, visualization techniques and their applications are often required to interpret the results of the analyses performed. Many tools and packages with different implementations can be used to visualize biological datasets, including genetic and genomic data [28, 29]. One popular web application framework widely used in various research fields is Shiny [30, 31] (https://www.rstudio.com/products/shiny/). Shiny is an open-source R package that offers the ability to develop interactive web applications (apps) with a dynamic user interface (UI) that can be run locally or deployed over the Internet. Shiny can be used in combination with Plotly's R graphing library [32] (https://plotly.com/r/) to create interactive web-based graphical representations of data, such as plots, charts, histograms, heatmaps, etc.

Integration of analysis and visualization functionalities into the same application or pipeline is an important approach used in various biomedical research areas, including genetics and genomics. There are some examples of pipelines that combine a comprehensive analysis of sequencing data and visualization capabilities. For example, Wang et al. [33] created the “CRISPR-DAV: CRISPR NGS data analysis and visualization pipeline,” which analyzes the CRISPR (clustered regularly interspaced short palindromic repeat) NGS (next generation sequencing) data and visualizes the analysis results. The pipeline itself is implemented in Perl and R and uses a set of common bioinformatics tools. Buza et al. [34] developed the “iMAP: an integrated bioinformatics and visualization pipeline for microbiome data analysis,” which performs the analysis of marker-based microbiome data using several publicly available tools and generates graphics and progress reports using various R packages and R-markdown. There are also several applications for PS and genetic relatedness analyses and the visualization of their results. However, as discussed later, these applications differ from the pipeline we have developed in terms of the types of analysis performed, functions offered to users, and their implementation.

In this study, we developed a PS and relatedness integrated pipeline, PSReliP, which analyzes and visualizes the PS and relatedness between individuals (samples) based on genome-wide genetic variant data. All analyses are performed at high speed using PLINK software in a sequential manner with programs and scripts written in-house. The Shiny web application allows users to interactively visualize the analysis results in a web browser. Herein, we described the structure of PSReliP, explained the functionality of its analysis and visualization stages and UI as well as demonstrated its application in genome-wide genetic variant data of rice varieties and Malawi cichlids.

## Implementation

### Pipeline structure and implementation

**Fig. 1:**
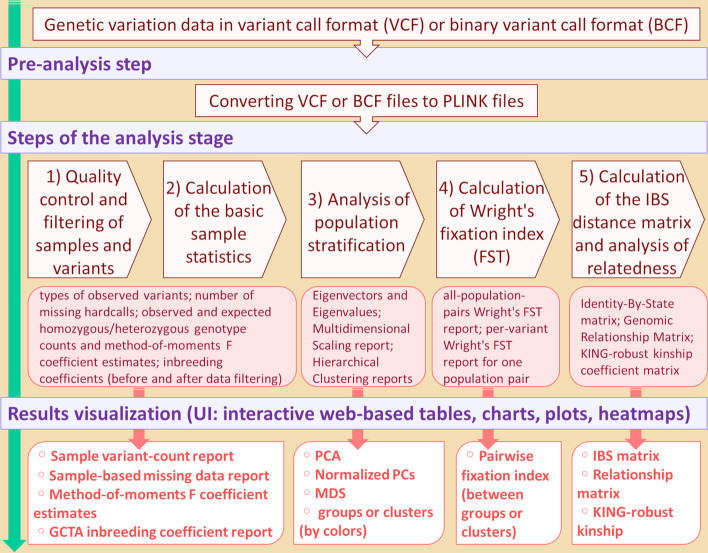
Conceptual overview of the pipeline structure. A schematic representation showing all major steps of the pipeline, with input and output data types and corresponding elements in the user interface. The first pre-analysis step in the pipeline is to convert variant call format (VCF) file or binary variant call format (BCF) file to PLINK files. The analysis phase performs all types of analysis, and the results of each step are visualized in interactive web-based tables, charts, plots, and heatmaps

The PSReliP pipeline combines analysis techniques with an interactive visualization of the analysis results. Figure 1 shows a conceptual overview of the pipeline structure with distinct steps and associated output files.

The proposed integrated pipeline can be divided into two stages: (1) the analysis stage, which includes a pre-analysis step and (2) the visualization stage. The analysis stage is performed by two bash shell scripts that are executed from the command line on Linux-based operating systems and take several arguments from the configuration file (see Additional file 2: Table S1 for details). The first step in the analysis stage is the conversion of the variant call format (VCF) or binary variant call format (BCF) files into PLINK format files, which are later used in the data analysis process. The main steps of the analysis stage of the pipeline are: (1) QC and filtering of samples and variants; (2) calculation of basic sample statistics, such as the types of observed variants, inbreeding coefficients, etc., which is performed before and after data filtering; (3) analysis of PS using PCA and MDS, and complete-linkage hierarchical clustering of samples based on the IBS distance matrix, if selected; (4) calculation of Wright's FST; (5) calculation of the IBS distance matrix and analysis of genetic relatedness by estimating the KING kinship coefficient matrix and GRM. All the steps are performed sequentially.

To visualize the results of the analyses, we created a web-based visualization stage for PSReliP. We implemented this stage using Shiny technology (https://shiny.rstudio.com/), which provides a dynamic and interactive UI, and developed the Shiny application, an interactive R-based web application. Users can run the created Shiny application locally in Rstudio or deploy it in two main ways: in their own Shiny Server or in the cloud: shinyapps.io (https://shiny.rstudio.com/tutorial/written-tutorial/lesson7/). Running the Shiny application produces interactive tables, plots, and charts of data and displays them through a web browser. In addition, the Shiny application allows the user to download the PLINK result files for evaluation and further use in other tools and software. All steps of the data analysis and visualization of our pipeline are elaborated in the Additional file 1: Note 1. In this study, we used the results of several runs of our pipeline applied to two datasets, which are described in the following sections. The time required to complete each of these runs is shown in Additional file 2: Table S2 (runs of the first shell script) and 3 (runs of the second shell script).

**Fig. 2:**
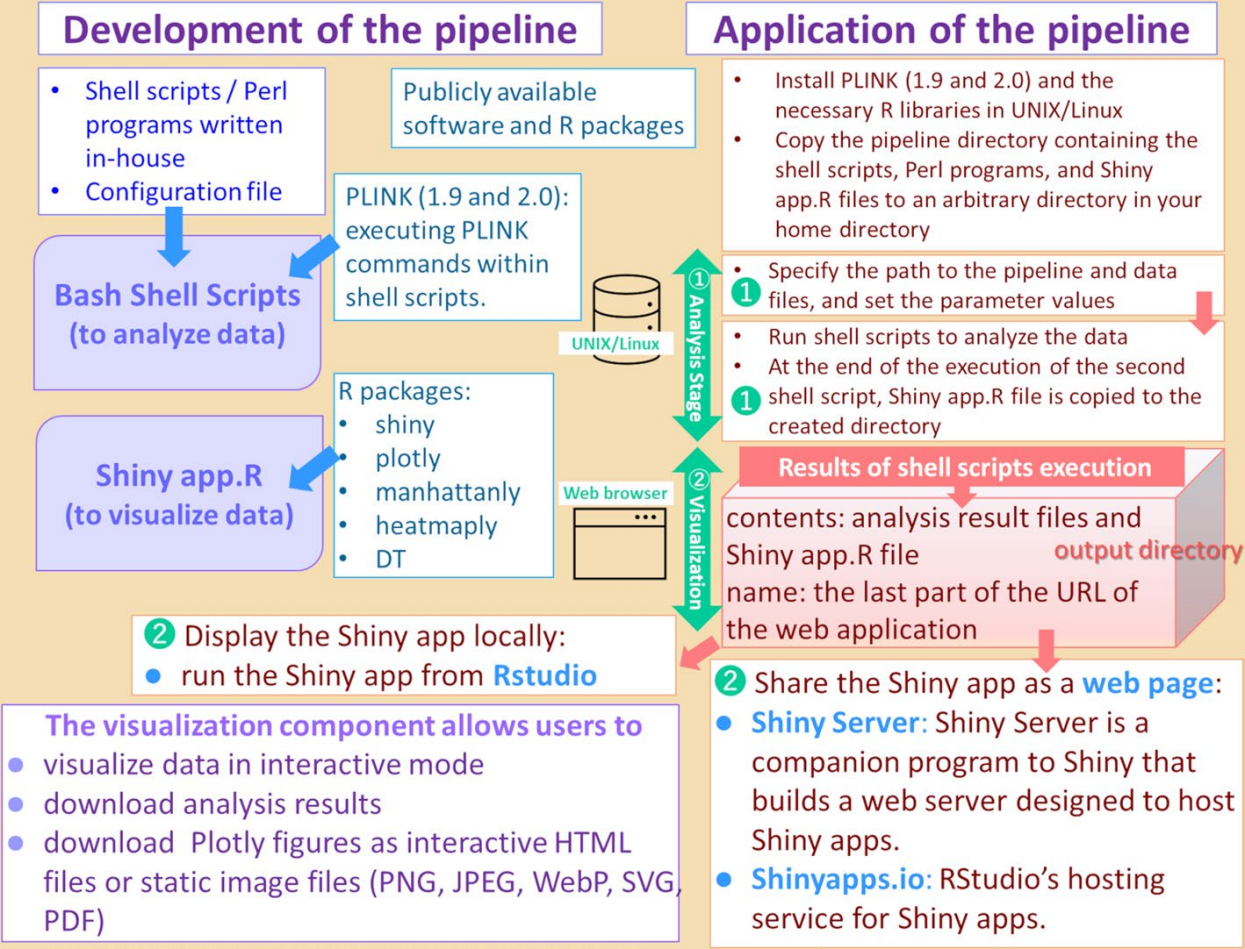
Implementation of the PSReliP pipeline. A schematic representation showing the required software and R packages used to develop the PSReliP pipeline and those that must be installed by the user before running the pipeline. The user must also edit the configuration file, which contains information about the pipeline installation directory, the working directory, input files, and parameter values used in the analysis and visualization processes. The created Shiny application can be viewed in several ways, such as sharing as a web page using Shiny Server or Shinyapps.io or running from RStudio Desktop

Figure 2 outlines the implementation of PSReliP and shows the major parts of the pipeline implemented in Shell, Perl, and R using the PLINK software and several publicly available R packages.

### User interface

To describe the functionality and application of our pipeline, along with the UI of its visualization stage, we used screenshots of the UI that appeared in the Google Chrome browser when PSReliP was run on a Shiny Server installed on our CentOS Linux. The data used in these screenshots were derived from the genome-wide genetic variant data of 143 worldwide rice samples registered in the Rice Annotation Project Database [35] (RAP-DB; https://rapdb.dna.affrc.go.jp) (details are described in the Results section). Additional details of the user interface shown in the following figures are described in Additional file 1: Note 2.

**Fig. 3:**
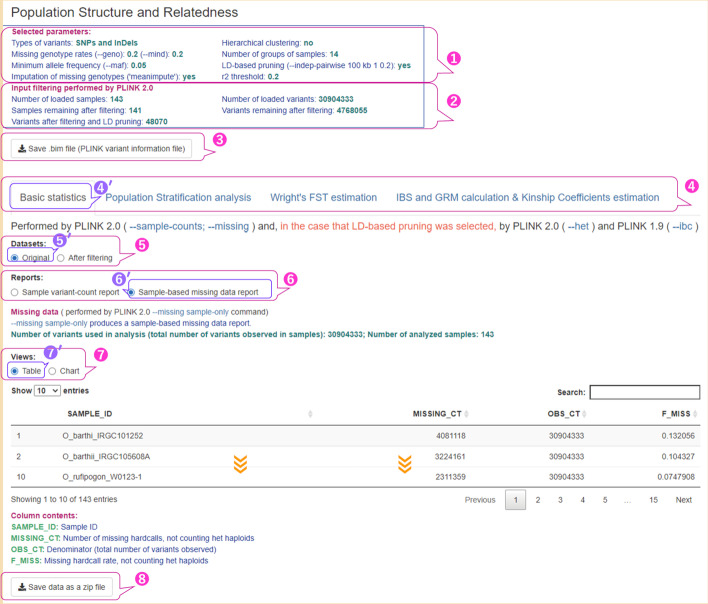
User interface for basic sample statistics with table representation of missing data. The parameters used are shown in Additional file 2: Table S3 (Run A). ① The parameters used in the analysis stage, which are specified in the configuration file; ② the numbers of analyzed samples, loaded variants, and variants remaining after filtering and LD pruning, which were calculated at the analysis stage; ③ a download button for the PLINK 1.9.bim file; ④ four tabs in the main menu of the user interface with the ‘Basic statistics’ tab selected; ⑤ a radio button with two values: ‘Original’ and ‘After filtering’, corresponding to the datasets displayed on this tab; ⑥ a radio button labeled ‘Reports’ whose values correspond to the types of reports displayed and its two values for the ‘Original’ dataset; ⑦ a radio button labeled ‘Views’ for the two types of data presentation such as ‘Table’ and ‘Chart’; ⑧ a button to download the results of the basic statistics analysis as a ZIP file. The Sample-based missing data report for 143 accessions of rice varieties is represented as an interactive table with sorting and searching features. The symbol prime (′) at the upper right of the numbers indicates selected items

**Fig. 4:**
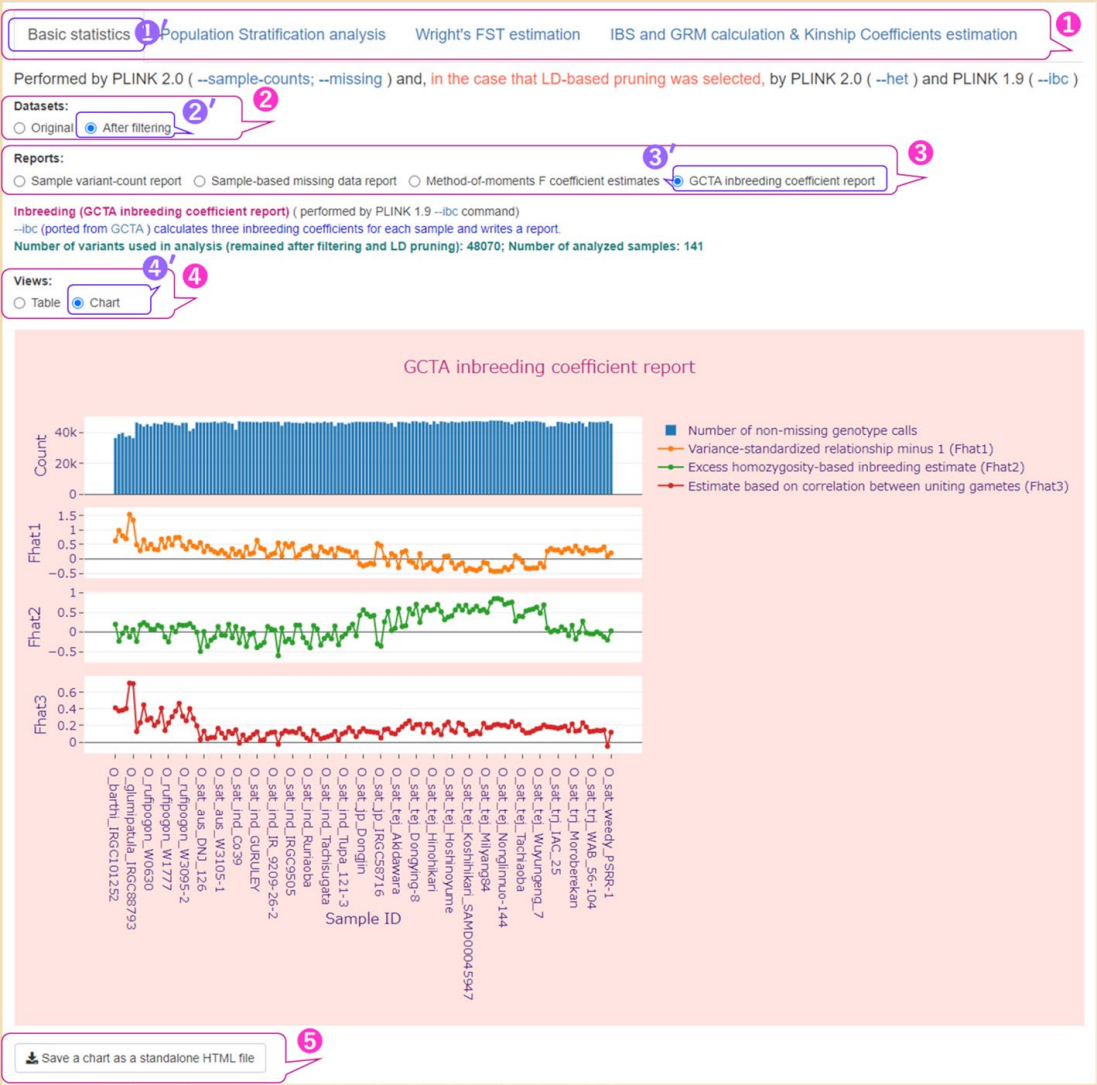
User interface for basic sample statistics with chart representation of inbreeding coefficients. The parameters used are shown in Additional file 2: Table S3 (Run A). ① Four tabs in the main menu of the user interface with the ‘Basic statistics’ tab selected; ② a radio button with two values: ‘Original’ and ‘After filtering’, corresponding to the datasets displayed on this tab; ③ a radio button labeled ‘Reports’ whose values correspond to the types of reports displayed and its four values for the ‘After filtering’ dataset; ④ a radio button labeled ‘Views’ for the two types of data presentation such as ‘Table’ and ‘Char’; ⑤ a button to download the displayed chart as a single standalone HTML file. The GCTA inbreeding coefficient report for 141 accessions of rice varieties is represented in the form of multiple interactive subplots, namely the bar chart for missing data and three scatter plots with lines for inbreeding coefficients. The symbol prime (′) at the upper right of the numbers indicates selected items

As described above, the pipeline visualization stage generates tables, plots, charts, and heatmaps to show the results of the analysis stage. Visualizing the analysis results in a user-friendly manner is important for interpretation and optimization of the analysis process. The main functionalities of the visualization stage are shown in Figs. 3 and 4. The parameters used in the analyses are listed in Additional file 2: Table S3 (Run A).

Figure 3 shows a screenshot of the web page shown when the Shiny app was accessed for the first time, with one exception: in this example, the [sample-based missing data reports ⑥′] value was selected after the page was loaded. Figures 3 ④ and 4 ① show the main menu of the PSReliP UI. The four tabs on this menu correspond to the types of analysis performed in our pipeline. The tabs are as follows: (1) ‘Basic statistics’; (2) ‘Population Stratification analysis’; (3) ‘Wright's FST estimation’; and 4) ‘IBS and GRM calculation & Kinship Coefficients estimation’. In both figures (Figs. 3 ④′ and 4 ①′), the ‘Basic statistics’ tab is selected, and the basic sample statistics are displayed. Figures 3 ⑤ and 4 ② show the radio button labeled ‘Datasets’ with two values: “original” and “after filtering.” These values correspond to the datasets displayed in this tab:

Original: the original dataset with no applied filters, which contains all the samples and variants included in the input VCF/BCF files. An example of this is shown in Fig. 3 ⑤′.

After filtering: all the filters specified in the configuration file were applied to the dataset.

An example of this is shown in Fig. 4 ②′.

The original PLINK result files can be downloaded as ZIP files so that users can evaluate the analysis results and further use them in other tools and software (Fig. 3 ⑧). The button labeled ‘Save the chart as a standalone HTML file’ shown in Fig. 4 ⑤ allows the user to download the displayed chart as a single standalone HTML file. In addition, using the features provided by Plotly, the user can export the displayed image from the browser as an image file in the format specified in the configuration file, such as PNG, JPEG, WebP, SVG, and PDF. Other UI features of our pipeline are illustrated in the Results section.

## Results

### Preparing data for case studies

To demonstrate the application of the proposed pipeline, validate its efficiency in assessing PS and CR, and illustrate its functionalities and capabilities, two case studies were conducted on rice varieties and Malawi cichlids. To prepare the data for these case studies, we first downloaded the sequencing data with associated metadata from the NCBI and EBI databases and then performed sequence alignment and variant calling using the procedure described in Additional file 1: Note 3. To create the dataset of rice varieties, we selected BioSample accessions of cultivars, landraces, and wild species, registered in the Rice Annotation Project Database [35] (RAP-DB, http://rapdb.dna.affrc.go.jp/) [36], with an average depth of sequencing coverage greater than 30. To create the dataset of Malawi cichlids, we selected BioSample accessions from BioProject PRJEB1254 and PRJEB15289, for which the sampling locations were recorded in the NCBI BioSample database. All raw sequencing reads were obtained from a previous study [37]. We compared the data obtained by running our pipeline with the data published in that article, which is discussed in the following subsections. Details of selecting BioSample accessions and reference genomes [38, 39], downloading nucleotide sequence data, and preparing genetic variant data are described in Additional file 1: Note 4. Accessions from the BioProject, BioSample, and European Nucleotide Archive databases are listed in Additional file 2: Tables S4 and S5.

### Results obtained in case studies

- Analysis of genetic variants of rice varieties

The results of the analyses performed five times using different filtering and pruning options (Additional file 2: Table S3) are described here.

**Fig. 5:**
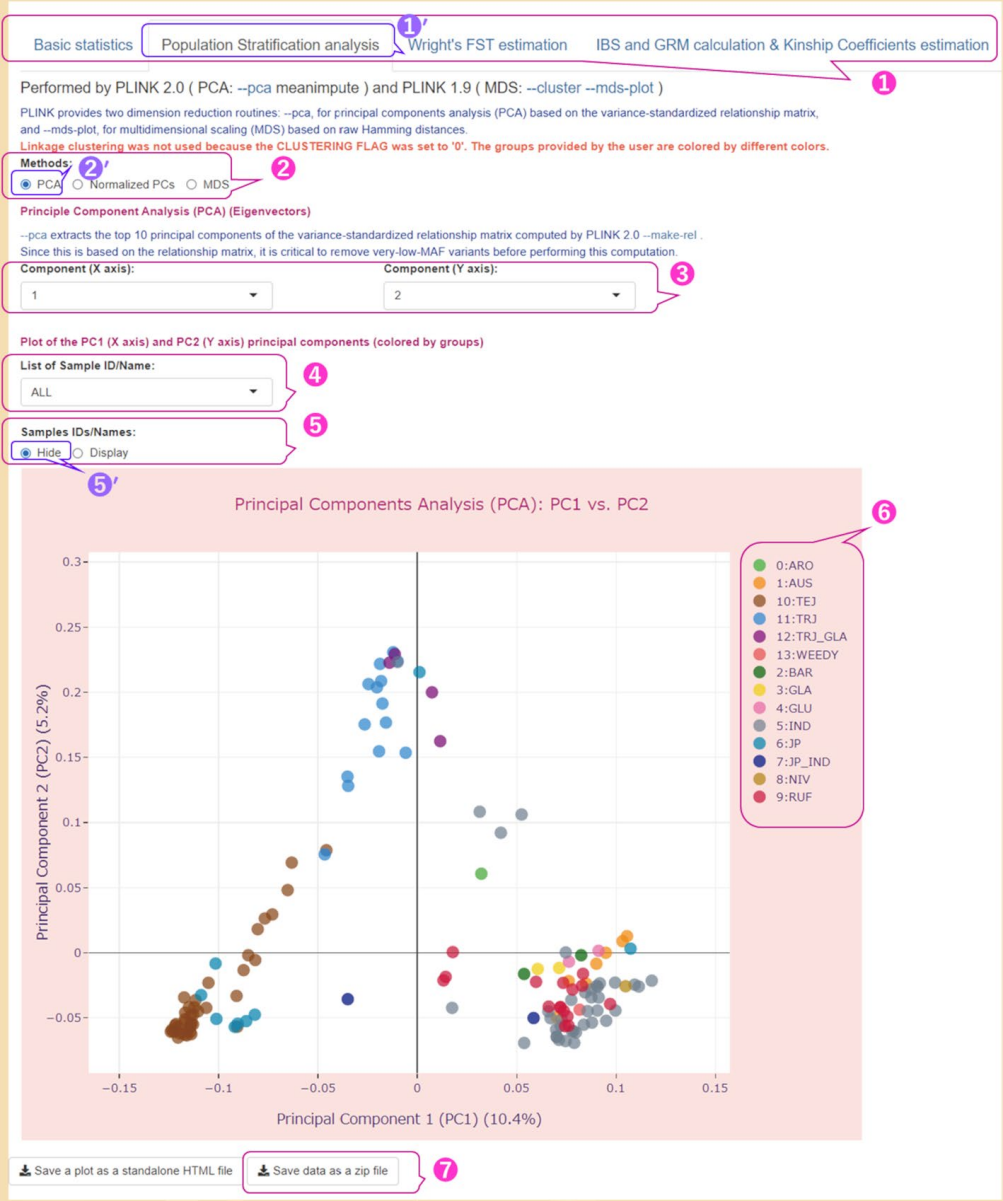
User interface for results of Population Stratification analysis with the two-component PCA plot. The parameters used are shown in Additional file 2: Table S3 (Run A). ① Four tabs in the main menu of the user interface with the ‘Population Stratification analysis’ tab selected; ② a radio button with a choice of three methods, namely PCA, Normalized PCs (each eigenvector is multiplied by the square root of its eigenvalue), and MDS; ③ two drop-down lists with values of principal components for which the PCA plot is drawn; ④ a drop-down list with the value ‘ALL’ or the ID/Name of the sample, which is displayed in a larger size compared to rest of the values; ⑤ a radio button with values ‘Hide’ and ‘Display’, which allows users to hide or show sample IDs/names; ⑥ the colors legend that matches groups/clusters with corresponding marker colors; ⑦ a button to download the results of the population stratification analysis as a ZIP file. The interactive two-component PCA plot displays the first and second principal components (PC1/PC2) for 141 accessions of rice varieties, with data point colors corresponding to 14 rice type groups. The symbol prime (′) at the upper right of the numbers indicates selected items

The four tabs on the main menu, corresponding to the types of analysis performed in our pipeline, are shown at the top of Fig. 5 (indicated by ①), of which the ‘Population Stratification analysis’ tab was selected (Fig. 5 ①′). The parameter values used in the PS analysis are listed in Additional file 2: Table S3 (Run A).

The scatter plot in Fig. 5 is an interactive 2-component PCA plot in which the first principal component (PC1) is represented by the horizontal axis and explains 10.4% of the variance, and the second principal component (PC2) is represented by the vertical axis and explains 5.2% of the variance. See Additional file 1: Note 5 for details on calculating the percentage of variance explained by each PC. The 2-component PCA plot for the other PCs can be drawn by selecting the corresponding components from the two drop-down lists, as shown in Fig. 5 ③. Users can highlight one of the samples on the PCA plot by selecting it from the drop-down list (Fig. 5 ④), and the selected sample will be shown to be larger than the others (Additional file 1: Fig. S1a). The interactive scatter plot can display annotation information, such as sample ID, PC values, and the group or cluster number to which the sample belongs, by hovering the mouse pointer over the markers (samples) in the scatter plot (Additional file 1: Fig. S1a, Run A in Additional file 2: Table S3). Users can “Hide” or “Display” the IDs or names of the samples by changing the checked value in the radio buttons (Fig. 5 ⑤, Additional file 1: Fig. S1b). In this figure, the ‘Hide’ value was checked (Fig. 5 ⑤′), and accordingly the names of the samples were hidden. Additionally, users can zoom in and out of the plot using Plotly's zoom functionality (Additional file 1: Fig. S1b, Run A in Additional file 2: Table S3).

In our pipeline, a PCA plot is a scatter plot that maps marker colors to a categorical variable (user-defined groups or clusters calculated using PLINK). Figure 5 ⑥ shows the color legend that matches the groups defined by us with the corresponding marker colors. We grouped 143 rice varieties into 16 groups based on the rice types (e.g., indica, aus, temperate japonica, tropical japonica, and aromatic) (Additional file 2: Table S6), similar to the way they were grouped in the RAP-DB. As mentioned earlier, after filtering by maximum missing genotype rates per-sample (—mind with a value of 0.2), the number of samples decreased to 141 and the number of groups decreased to 14 (“MER: Oryza meridionalis” and “PUN: Oryza punctata" were excluded) (Fig. 5). The japonica varieties (JP: Oryza sativa Japonica Group, TEJ: Oryza sativa temperate japonica subgroup, TRJ: Oryza sativa tropical japonica subgroup) and indica varieties (IND: Oryza sativa Indica Group, AUS: Oryza sativa aus subgroup) were separated from each other along PC1, whereas the TRJ and TEJ groups as well as the TRJ group and indica varieties were separated along PC2 (Fig. 5). The same can be observed in the MDS plot (Additional file 1: Figs. S2 and S3, Run A in Additional file 2: Table S3). Plotly provides functions to show/hide data from each group individually by clicking on corresponding legend items. We used this feature on all charts and plots in our pipeline.

For a more complete overview of the results of the PCA and MDS analyses, along with the projections of the samples on the plane defined by the first two PCs, plots of PC1 and PC3 are often used. Additional file 1: Fig. S4 shows plots of PC1 and PC3 obtained from the same analysis, as illustrated in Fig. 5 (Run A in Additional file 2: Table S3). PC3 explains 3.2% of the variance. Samples from groups such as BAR (Oryza barthii), GLA (Oryza glaberrima), and GLU (Oryza glumaepatula) as well as some samples from the RUF (Oryza rufipogon) group, which are indicated in the figure by an oval, were separated along PC3 from that of other groups, including the Oryza sativa groups mentioned above (Additional file 1: Fig. S4).

**Fig. 6:**
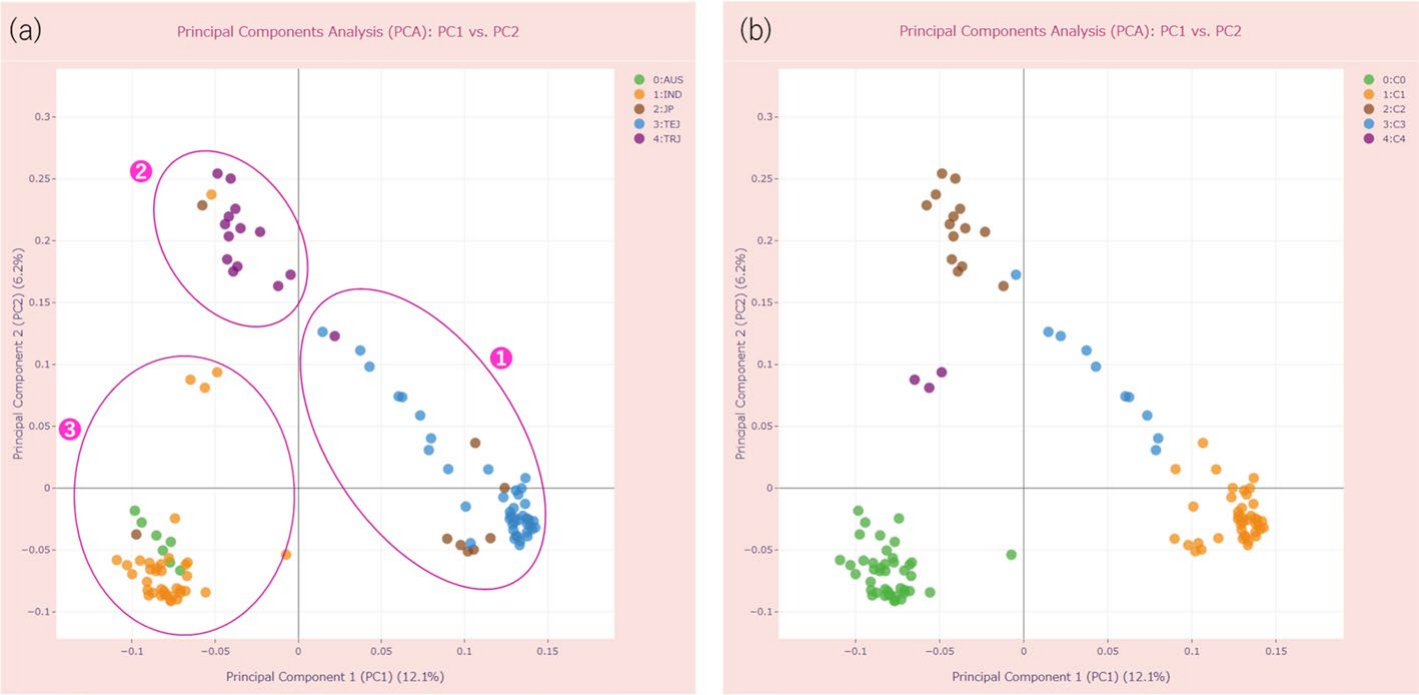
An illustration showing two ways of dividing samples into groups that are performed in PSReIP. **a**, **b** The two-component PCA plots for the first and second principal components (PC1/PC2) for 110 accessions of rice varieties from five groups: JP, TEJ, TRJ, IND, and AUS. **a** An example of using user-defined groups. The parameters used are shown in Additional file 2: Table S3 (Run C). The colors of the data points correspond to these 5 groups of rice type. ① Most samples from the TEJ group; ② almost all samples from the TRJ group; ③ almost all samples from the indica varieties, which include the IND and AUS groups. **b** An example of using complete-linkage hierarchical clustering performed by PLINK 1.9. The parameters used are shown in Additional file 2: Table S3 (Run E). The colors of the data points correspond to the 5 calculated clusters

To analyze PS and CR in japonica and indica varieties used in our case study, we selected samples belonging only to five groups (JP, TEJ, TRJ, IND, and AUS) and performed the analysis stage. The resulting PCA plots are shown in Fig. 6a (Run C in Additional file 2: Table S3) and Fig. 6b (Run E in Additional file 2: Table S3).

**Fig. 7:**
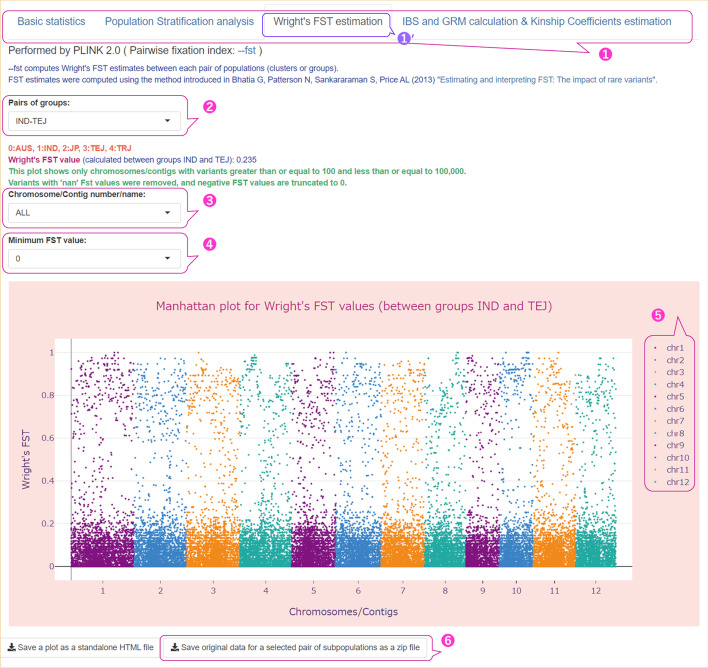
User interface for results of FST estimation with the Manhattan plot of FST values for variants. The parameters used are shown in Additional file 2: Table S3 (Run C). ① Four tabs in the main menu of the user interface with the ‘Wright's FST estimation’ tab selected; ② a drop-down list for selecting a pair of subpopulations to be shown on the Manhattan plot; ③ a drop-down list with the value ‘ALL’ or the name of the chromosome/contig containing the variants ≥ 100 and ≤ 100,000; ④ a drop-down list with range of FST values (0–0.9) to filter the data displayed in the Manhattan plot; ⑤ the legend colors on the plot corresponding to the chromosome number; ⑥ a button to download the results of the Wright's FST estimation as a ZIP file. The interactive Manhattan plot showing Wright's FST values for variants on all chromosomes that were calculated between the IND and TEJ rice groups. The symbol prime (′) at the upper right of the numbers indicates selected items

As described above, during the analysis stage of our pipeline, the PLINK—fst command is executed, and the results are visualized in the ‘Wright's FST estimates’ tab (Fig. 7 ①′, Additional file 1: Fig. S5) of the tabs panel (Fig. 7 ①). The parameter values used in FST analysis are listed in Additional file 2: Table S3 (Run C).

Users can select one of the pairs of subpopulations (groups or clusters of samples) by choosing the pair from the drop-down list (Fig. 7 ② and Additional file 1: Fig. S5 ①), and Wright's FST value between the pairs of selected subpopulations (pairwise FST) is displayed in the text box immediately below this drop-down list. In this example, this value was 0.235 for the IND and TEJ groups.

The PLINK—fst command with the 'report-variants' modifier calculates the per-variant FST estimates, which is used in our pipeline if the number of groups/clusters is ≤ 5 (to control the output size). The FST values for each variant between pairs of the selected subpopulations are shown in the Manhattan plot (Fig. 7 and Additional file 1: Fig. S5). Variants with 'nan' Fst values were removed from the FST plot, and negative FST values were set to zero. For a particular variant, an FST value near 1 indicated that each of the two populations was fixed for a different allele at that locus, similar to the variant shown in Additional file 1: Fig. S5 ④. The legend colors in the plot (Fig. 7 ⑤) correspond to chromosome numbers. The download button labeled ‘Save original data for a selected pair of subpopulations as a zip file’ (Fig. 7 ⑥) allows the user to download files obtained with the PLINK—fst command and containing FST estimates between the two selected subpopulations.

To illustrate how the FST values depend on the variants used in the analysis, we ran our pipeline on the same input subset (Run C in Additional file 2: Table S3) without LD-based pruning and with the—maf parameter of 0.01 (Run D in Additional file 2: Table S3). For comparison, we placed the HUDSON_FST values (between-population FST estimates) obtained from the two runs in Table 1, which shows that the FST values are significantly lower in the LD pruned data, and this result is consistent with those presented in the scientific literature, which is discussed in the Discussion section.

**Table 1:** Pairwise Hudson's FST between groups in the dataset of genetic variants of rice varieties

POP1	POP2	HUDSON_FST^a^	HUDSON_FST (LD pruned variants set^b^)
AUS	IND	0.34	0.094
AUS	JP	0.569	0.202
AUS	TEJ	0.72	0.29
AUS	TRJ	0.615	0.202
IND	JP	0.46	0.161
IND	TEJ	0.605	0.235
IND	TRJ	0.486	0.15
JP	TEJ	0.124	0.053
JP	TRJ	0.259	0.149
TEJ	TRJ	0.412	0.203

^a^Data were not pruned for LD and the—maf value was set to 0.01 (Run D in Additional file 2: Table S3)

^b^The parameters used are listed in Additional file 2: Table S3 (Run C)

**Fig. 8:**
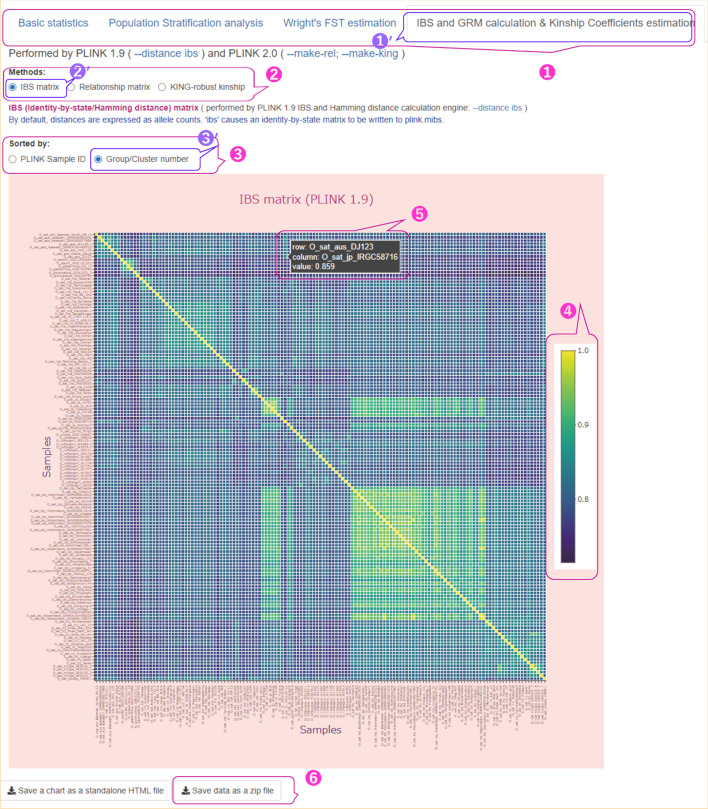
User interface for results of IBS, GRM, and Kinship coefficient calculations with heatmap of IBS displayed. The parameters used are shown in Additional file 2: Table S3 (Run A). ① Four tabs in the main menu of the user interface with the ‘IBS and GRM calculation & Kinship Coefficients estimation’ tab selected; ② a radio button for choosing any of the three methods, namely the IBS matrix calculation, the Genomic Relationship Matrix calculation, and the KING-robust kinship estimation; ③ a radio button to select the order of the samples, which can be the same as in the matrix obtained by the corresponding PLINK command (‘PLINK Sample ID’) or can be remade according to the groups/clusters to which the samples are assigned (‘Group/Cluster number’); ④ a gradient color bar that maps colors to their corresponding values; ⑤ the corresponding value for pairs of samples and their IDs/names displayed by hovering the mouse over the colored square; ⑦ a button to download the results of the IBS, GRM, and kinship calculation as a ZIP file. The interactive heatmap represents the genetic similarity between samples, namely the IBS matrix calculated for 141 accessions of rice varieties, in which the samples are ordered by ‘Group/Cluster number’. The symbol prime (′) at the upper right of the numbers indicates selected items

A Fig. 8 shows an example of the genetic similarity between individuals (samples) and the genetic relatedness between them (Run A in Additional file 2: Table S3).

Users can display the results of these analyses by selecting the tab ‘IBS and GRM calculation and Kinship Coefficients estimation’ (Fig. 8 ①′) on the main menu (Fig. 8 ①). For this type of analysis, we prepared three methods (Fig. 8 ②): IBS matrix calculation (Fig. 8 ②′), GRM, and KING-robust kinship estimation. The results of these three types of calculations are displayed on interactive heatmaps, where samples can be ordered in two ways, ‘PLINK Sample ID’ and ‘Group/Cluster number’ (Fig. 8 ③). The list of sample IDs/Names on the heatmap can be in the same order as in the matrix derived from the corresponding PLINK command, or samples can be reordered according to the groups/clusters to which they are assigned (Fig. 8 ③′). The gradient color bar in the heatmap (Fig. 8 ④) maps the colors to their corresponding values. The individual values for the two samples and the IDs/Names for these samples are displayed when the mouse is hovered over the colored square (Fig. 8 ⑤). Additional file 1: Fig. S6 shows a heatmap of the GRM (Additional file 1: Fig. S6 ①) for the same data, as shown in Fig. 8 (Run A in Additional file 2: Table S3). The samples are also ordered by ‘Group/Cluster number’ (Additional file 1: Fig. S6 ②).

Scientific literature suggests that pruning data based on LD values is an important step for IBS and GRM calculations (see Discussion section). To illustrate how the values of IBS and GRM depend on the variants used in the analysis, we ran our pipeline on the same input subset (Run A in Additional file 2: Table S3) without using LD-based pruning and with the—maf parameter of 0.01 (Run B in Additional file 2: Table S3). The resulting IBS matrix (Additional file 1: Fig. S7 ①), reordered by the ‘Group/Cluster number’ (Additional file 1: Fig. S7 ②), is shown in Additional file 1: Fig. S7. As can be observed from the two heatmaps (Fig. 8 and Additional file 1: Fig. S7), without LD-based pruning, the overall values of the IBS matrix are higher, as in the example of the same pair of samples (Fig. 8 ⑤ and Additional file 1: Fig. S7 ③).

Unlike the calculation of IBS and GRM, LD-based pruning is not recommended for estimating KING-robust kinship coefficients (see Discussion section). Additional file 1: Fig. S8 shows an example of a heatmap of KING-robust kinship coefficients (Additional file 1: Fig. S8 ①) for data that were not pruned for LD, and the—maf value was set to 0.01 (Run B in Additional file 2: Table S3). The samples on the heatmap were ordered by ‘Group/Cluster number’ (Additional file 1: Fig. S8 ②). Note that the KING kinship coefficients are scaled so that duplicate samples have a kinship of 0.5, rather than 1 (Additional file 1: Fig. S8 ③). In this heatmap, most of the individual pairs had a kinship coefficient of 0, and only a few pairs had a kinship coefficient > 0.25, such as a pair of accessions of the Koshihikari variety with a KING kinship coefficient of 0.358 (Additional file 1: Fig. S8 ④). As described above, to explore CR between individuals, we prepared GRM and the kinship matrix with pairwise KING kinship coefficients, so that users can choose between them depending on the objectives of the study and the type of downstream analysis.

- Analysis of genetic variants of Malawi cichlids

In this subsection, we present the results of the analysis of the dataset containing the genetic variants of Malawian cichlids. These results can be viewed in the following tables: ‘Basic statistics,’ ‘Population Stratification analysis,’ and ‘Wright's FST estimation.’ We ran our pipeline multiple times using different filtering and pruning options (Additional file 2: Table S3 Dataset of genetic variants of Malawi cichlids). The parameters used in Runs F and G differed in the number of groups, whereas the parameters used in Run H differed from those used in Run I by using LD-based pruning and values of the—maf parameter.

In the stacked bar chart of a ‘Sample variant-count report’ for the original dataset (Run F in Additional file 2: Table S3), most of the observed variants belonged to the class of ‘Hom-REF genotype’ (homozygous reference allele; reference: M_zebra_UMD2a) (blue color), and only a small number of observed variants belong to other classes, such as ‘Hom-ALT SNP’ (orange), ‘Het. SNP genotype’ (green), and ‘diploid non-SNP variant’ (red) (Additional file 1: Fig. S9). This result is consistent with that observed in a previous study showing that the genetic diversity in cichlid fish species is low [37]. Ten samples had more variants other than the ‘Hom-REF genotype’ class than the other samples. All were samples of the outgroup Astatotilapia species.

In the “Basic statistics” tab, the inbreeding coefficient (F) of each sample estimated based on the expected and observed individual heterozygosity can be shown in the grouped bar chart and line plot (Additional file 1: Fig. S10, Run F in Additional file 2: Table S3). These multiple subplots can be displayed by selecting the “Method-of-moments F coefficient estimates” report for the “After filtering” dataset on the “Basic statistics” tab. For most samples, the observed number of heterozygous genotypes was significantly lower than the expected number of heterozygous genotypes, and these values were approximately equal in few samples (Additional file 1: Fig. S10). Conversely, the observed number of homozygous genotypes was higher than expected in most samples. The low expected heterozygosity found in this analysis indicates high homozygosity and low genetic diversity in Malawi cichlids [37].

Given the information on sampling locations, we divided the samples into 17 groups according to their geographic locations (see Additional file 2: Table S7 for details). We also grouped the samples into seven eco-morphological groups in the same way as described in the article [37] and in an additional “outgroup Astatotilapia” (see Additional file 2: Table S7 for details). By applying these two sets of groups, we analyzed the same input dataset using the same filtering and pruning parameters. The PCA plots for PC1 and PC2 obtained from these runs are shown in Additional file 1: Fig. S11 (Run F in Additional file 2: Table S3, 17 groups) and Additional file 1: Fig. S12 (Run G in Additional file 2: Table S3, eight groups). The groups separated from each other by the first and second PCs correlated well with the eco-morphological groups indicated by colors (Additional file 1: Fig. S12). In contrast, positions in the PCA plot and sampling locations were not correlated, possibly because the species used in the case study belonged to different genera and were genetically diverged despite living in the same region (Additional file 1: Fig. S11). However, when examining individuals only from the species Astatotilapia calliptera of the genus Astatotilapia, the PCA plot showed some association between genetic similarity among these individuals and sampling locations (Additional file 1: Fig. S13). The PCA plot shown in Additional file 1: Fig. S13 is an enlarged view of the region shown in Additional file 1: Fig. S11, indicated by an oval in the upper-right corner. All samples from this region belonged to the Astatotilapia calliptera species, as shown in Additional file 1: Fig. S12 (indicated by ①).

It is interesting to note that individuals from the A. calliptera species group from the Lake Malawi catchment (green) are closer to individuals from the mbuna group (red) compared to those from the “outgroup Astatotilapia” (orange) that belonged to the same species of A. calliptera but were sampled from outside Lake Malawi (Additional file 1: Fig. S12). This is in agreement with the observations of Malinsky et al. [37].

**Fig. 9:**
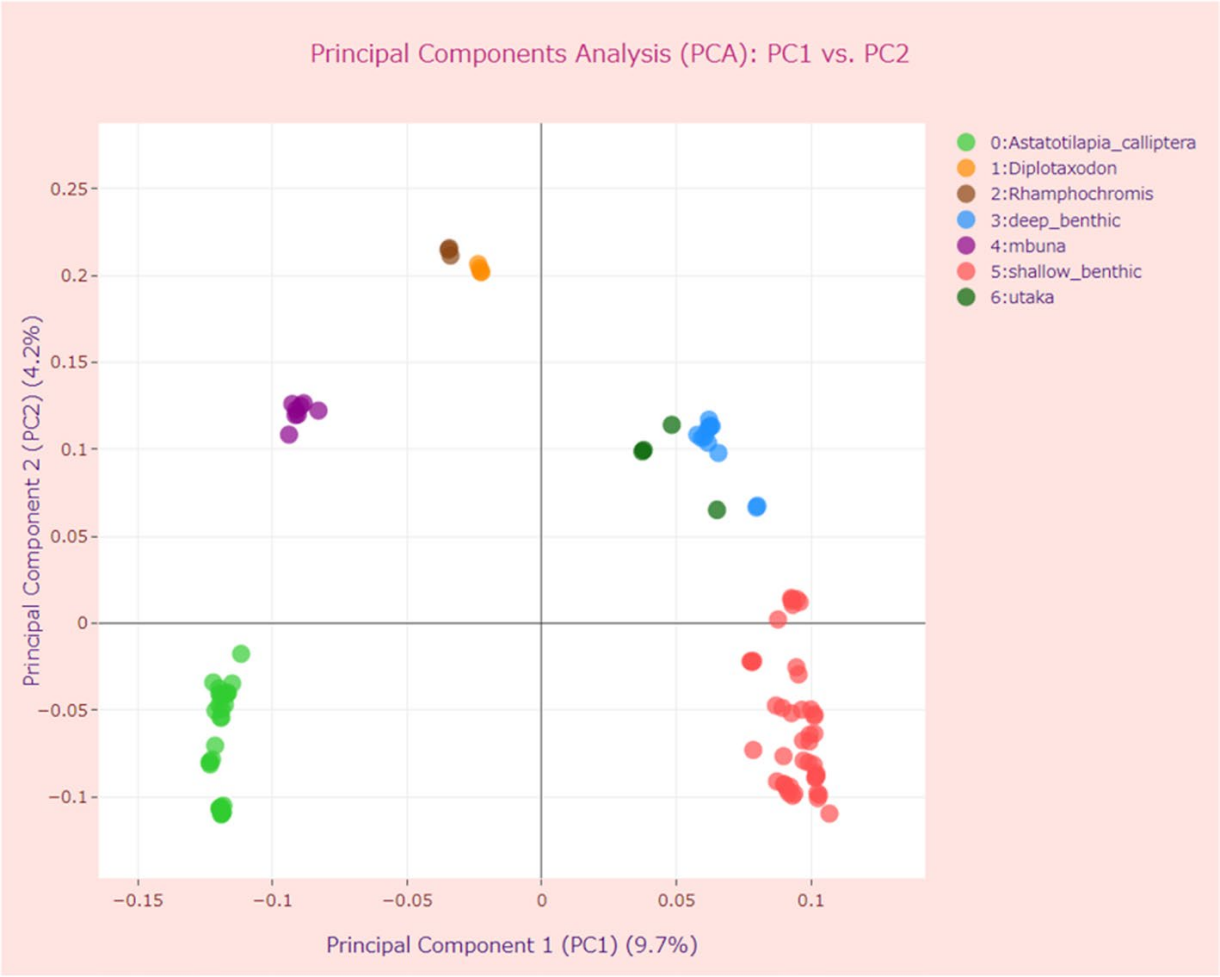
Principal Components Analysis (PCA) of genome-wide genetic variant data of Malawi cichlids. The parameters used are shown in Additional file 2: Table S3 (Run H). The interactive two-component PCA plot displaying the first and second principal components (PC1/PC2) for 109 accessions of Malawi cichlids, with data point colors corresponding to seven eco-morphological groups

We ran PSReliP on the samples without “outgroup Astatotilapia” (Additional file 1: Fig. S12 ②) and compared the results of PCA with those of Malinsky et al. [37] (Fig. 9, Run H in Additional file 2: Table S3).

The results were in agreement in terms of the distribution of groups relative to each other and to both axes of the PCs, the values of eigenvectors, and the percentage of variance explained by each component. For example, PC1 explained 9.7% of the variance in our case (7.9% in Malinsky et al. [37]), whereas PC2 explained 4.2% of the variance in both cases. We also created pairwise PCA plots of the top 3–10 PCs for 109 accessions of Malawi cichlids (Additional file 1: Fig. S14a–d) and found that our results were similar to those reported by Malinsky et al. [37].

A comparison of the FST values obtained from the two runs (Runs H and I in Additional file 2: Table S3) with those presented by Malinsky et al. [37] showed that the FST values between the A. calliptera group and other groups shown in Malinsky et al. [37] were between the values obtained from the two runs (Table 2). Regarding the FST values between other groups, the values presented by Malinsky et al. [37] were slightly lower than the values we obtained for the data pruned for LD (Run H in Additional file 2: Table S3).

**Table 2:** Pairwise Hudson's FST between groups in the dataset of genetic variants of Malawi cichlids

POP1	POP2	HUDSON_FST^a^	HUDSON_FST (LD pruned variants set^b^)
Astatotilapia_calliptera	deep_benthic	0.319	0.216
Astatotilapia_calliptera	Diplotaxodon	0.416	0.338
Astatotilapia_calliptera	mbuna	0.278	0.217
Astatotilapia_calliptera	Rhamphochromis	0.507	0.451
Astatotilapia_calliptera	shallow_benthic	0.308	0.197
Astatotilapia_calliptera	utaka	0.350	0.250
deep_benthic	Diplotaxodon	0.283	0.241
deep_benthic	mbuna	0.262	0.230
deep_benthic	Rhamphochromis	0.416	0.359
deep_benthic	shallow_benthic	0.103	0.081
deep_benthic	utaka	0.135	0.106
Diplotaxodon	mbuna	0.354	0.317
Diplotaxodon	Rhamphochromis	0.477	0.402
Diplotaxodon	shallow_benthic	0.300	0.261
Diplotaxodon	utaka	0.312	0.260
mbuna	Rhamphochromis	0.461	0.425
mbuna	shallow_benthic	0.258	0.224
mbuna	utaka	0.290	0.253
Rhamphochromis	shallow_benthic	0.417	0.360
Rhamphochromis	utaka	0.456	0.390
shallow_benthic	utaka	0.143	0.118

^a^Data were not pruned for LD and the—maf value was set to 0.01 (Run I in Additional file 2: Table S3)

^b^The parameters used are listed in Additional file 2: Table S3 (Run H)

Thus, we conclude that the results obtained by our pipeline are consistent with those shown in the original study, which confirms the ability of our pipeline to perform reliable analyses.

## Discussion

Understanding PS and CR is important in many application areas, including population genetics research, GWAS, and GS. There are several examples in the literature in which tools or pipelines have been created to visualize PC and/or CR after performing appropriate analyses based on genetic variant data. Steinig et al. [40] have developed “NETVIEW P” that is a comprehensive implementation of NETVIEW (the network analysis and visualization pipeline, Neuditschko et al. [41]) in Python. NETVIEW P combines data QC with the construction of population networks that can efficiently visualize the genetic structure within and between populations, including relationships and structure at the family level. In the NETVIEW P tool, the parameters and options can be set via the command line, and the input formats are the PED and MAP files from the PLINK software or a simple SNP matrix. The final network visualizations were based on the layouts provided by Cytoscape (https://cytoscape.org/), and the final network files were loaded into a compatible visualization platform.

Another example of such tools is “KinVis” [42], which was designed to analyze GWAS input data to identify relatedness. The KinVis tool was developed as an R-Shiny application; in this respect, it is similar to the implementation of the visualization stage of our pipeline. However, KinVis differs from our pipeline in terms of the types of analyses performed, their implementation, and the functions offered to the users.

In contrast to the tools mentioned above, in our pipeline, QC and a wide range of analyses such as PCA, MDS, Wright's FST estimation, calculation of IBS and GRM, inbreeding and kinship coefficient estimation, and some other calculations for the analysis process are executed in the same single workflow using PLINK software as well as the in-house shell scripts and PERL programs for data pipelining.

However, combining such diverse analyses in one workflow has its own challenges because different filtering criteria and, accordingly, different sets of genetic variants are considered optimal for different analyses. For example, for some analyses used in GWAS, such as IBD Estimation, inbreeding coefficient estimation (f), and PCA, better results can be obtained by selecting and analyzing markers that are not in LD with each other [43, 44]. Hence, LD-based pruning is effective in these types of analyses.

However, LD-based pruning is not recommended for some kinship estimation methods, such as the estimation of KING-robust kinship [13].

Regarding FST estimation, an inappropriate choice of criteria for the selection of genetic variants can lead to different FST values, particularly in cases where the population harbors a large number of rare variants [22], and LD-based pruned data underestimates FST values [43].

With our pipeline, users can set criteria for filtering and pruning samples and genetic variants by modifying parameters and performing the analysis multiple times, thereby overcoming these challenges. Visualization of the results of the various analyses is also performed by a single interactive web application implemented in R using the Shiny, Plotly, and other packages. Using the interactive features of Plotly inside a Shiny app allows researchers and developers to quickly create various visualizations commonly used in bioinformatics, such as dendrograms, heatmaps, Manhattan, volcano plots, etc., and publish or share them as an interactive web application.

The visualization stage of our pipeline allows users to view detailed analysis results in a web browser in the form of interactive tables, plots, and charts, which helps them quickly understand and interpret their data and decide which approaches are best for downstream analysis.

In this paper, we described the application of PSReliP in genome-wide genetic variant data of rice varieties and Malawi cichlids. While sample sizes of the two case studies were relatively small, PSReliP pipeline can handle larger dataset and data created in the pipeline can be directly used for downstream analysis such as GWAS (Additional file 1: Note 6 and Additional file 1: Figs. S15–S19). Our pipeline not only allows users to analyze PS and CR in the target population but also helps conduct further molecular genetics studies.

## Conclusions

In this study, we developed a computational and visualization pipeline that enables users to infer PS and estimate CR at high speeds and to visualize processed input and output data interactively. To build the pipeline, existing software and R packages, such as PLINK, Shiny, Plotly, and others, were used together with our self-written programs and scripts. In addition, various parameters were prepared using PSReliP for analysis and visualization processes. Therefore, it is expected that by changing the parameters and repeatedly performing the corresponding analysis, it will be easier to select a suitable set of variants and samples and the most appropriate PCA and kinship coefficients for further use in downstream analyses, including GWAS and GS. To facilitate this process, PSReliP provides the functionality to download all the original PLINK results as ZIP files, in addition to the ability to download tables, plots, and charts of the analyzed data as image files. To validate PSReliP, investigate its performance, and illustrate its various features, we conducted case studies on rice and Malawi cichlid accessions. The findings from these case studies demonstrate the ability of the proposed pipeline to correctly estimate PS and CR in the datasets provided. Designed as an integrated platform for data analysis and visualization, we hope that this pipeline becomes a useful tool for analyzing genome-wide genetic variant data (single-nucleotide polymorphisms and small insertions and deletions) to identify PS and CR and help avoid potential problems associated with them that may arise in further analysis.

## Availability and requirements

Project name: PSReliP.

Project home page: https://github.com/solelena/PSReliP

Operating system(s): Linux-based operating systems.

Programming language: Bash, R, Perl.

Tool: PLINK 1.9: 2 Apr 2022 or later, PLINK 2.0: 24 Oct 2022 or later.

R and R packages: R (3.6 +), shiny (1.4.0.2+), plotly (4.9.2.1+), manhattanly (0.2.0+), heatmaply (1.1.0+), ggplot2 (3.3.0+), DT (0.16+), stringr (1.4.0).

Web browsers: Google Chrome, Mozilla Firefox, and Microsoft Edge.

License: GNU GPL v3.0

Any restrictions to use by non-academics: license needed.

## Supplementary Information

**Additional file 1**. **Notes 1-6.** Detailed information about the datasets used, all stages of data analysis and visualisation, their implementation and the user interface.

**Additional file 2**. **Tables S1-S7.** Detailed information about the parameters used in the pipeline configuration file, the parameter values and the required time to analyze example datasets, and the accession numbers for data used in the case studies.

## Data Availability

The documentation and the source code of PSReliP are available at https://github.com/solelena/PSReliP. Detailed instructions on installing the pipeline can be found in the main README file of the PSReliP repository on GitHub. To prepare the datasets on rice varieties and Malawian cichlids used as input data for PSReliP, the raw sequence data in FASTQ format were downloaded from the European Nucleotide Archive (ENA) database by accession number, as listed in Additional file 2: Tables S4 and S5. In these tables, the ‘BioSample accession’ represents the BioSample accession numbers from the BioSample database (https://www.ncbi.nlm.nih.gov/biosample/); the ‘BioProject accession’ represents the BioProject accession numbers from BioProject (https://www.ncbi.nlm.nih.gov/bioproject/); and the ‘Run accession’ represents the SRA accession numbers from the Sequence Read Archive (SRA) based on the source database (SRA, European Bioinformatics Institute (EBI), or DNA Data Bank of Japan (DDBJ)). The SRA run accessions that were used for to create the dataset of rice varieties: DRR018360, DRR018358, DRR018356–DRR018357, DRR018359, DRR018355, DRR008446–DRR008447, DRR001185, DRR000719–DRR000720, DRR003661, DRR003656–DRR003657, DRR003660, DRR003652, DRR003655, DRR003650–DRR003651, DRR003659, DRR003653–DRR003654, DRR003648–DRR003649, DRR003658, DRR004451–DRR004453, DRR125602, DRR054198, DRR054216, DRR099981–DRR099990, DRR093954–DRR093956, DRR093959, DRR093961–DRR093970, DRR092498, DRR092500, DRR128976–DRR128981, ERR2240123–ERR2240128, ERR2241054–ERR2241058, ERR2241060, ERR2242620–ERR2242626, ERR2245512–ERR2245513, ERR2245515–ERR2245520, ERR2245522–ERR2245525, ERR2245527–ERR2245529, ERR2245533–ERR2245540, ERR2245542–ERR2245544, ERR2245546–ERR2245557, ERR157947, ERR161560, ERR161567–ERR158568, ERR161564, SRR1614244, SRR546420, SRR546695, SRR921498, SRR921505, SRR923809, SRR925387, SRR933669, SRR1016473–SRR1016474, SRR1016489–SRR1016491, SRR3056468, SRR1328207, SRR1328218, SRR1328220, SRR1328233, SRR1328254, SRR1450213–SRR1450215, SRR1450217, SRR1450219, SRR1450197–SRR1450198, SRR1528301, SRR1528330, SRR1528440, SRR1528449, SRR1712585, SRR1712645–SRR1712647, SRR1712649, SRR1712651, SRR1712653, SRR1712656, SRR1712898–SRR1712901, SRR1712903–SRR1712907, SRR1712909–SRR1712910, SRR1712953, SRR1712964–SRR1712970, SRR1712971–SRR1712981, SRR3056114, SRR3056466, SRR3056278, SRR3133641, SRR5880534, SRR6166428. The SRA run accessions that were used for to create the dataset of Malawi cichlids: ERR266450–ERR266461, ERR266464–ERR266485, ERR266488–ERR266493, ERR266496–ERR266498, ERR266502, ERR266505, ERR266508–ERR266509, ERR266511, ERR266513, ERR271651, ERR271655, ERR271658–ERR271659, ERR271663, ERR271666, ERR271669, ERR271670, ERR271672, ERR271674, ERR271677–ERR271678, ERR271680, ERR271682, ERR295124–ERR295132, ERR299198–ERR299215, ERR303339–ERR303340, ERR303342–ERR303343, ERR303345–ERR303346, ERR303348–ERR303349, ERR303351–ERR303352, ERR303354–ERR303355, ERR303357–ERR303358, ERR303360–ERR303361, ERR303363–ERR303364, ERR303366–ERR303367, ERR303369–ERR303370, ERR303372–ERR303373, ERR303375–ERR303376, ERR303378–ERR303379, ERR303381–ERR303382, ERR303384–ERR303385, ERR303387–ERR303388, ERR303390–ERR303391, ERR303393–ERR303394, ERR303396–ERR303397, ERR303399–ERR303400, ERR303402–ERR303403, ERR303405–ERR303406, ERR303408–ERR303409, ERR315227–ERR315228, ERR315230–ERR315231, ERR315233–ERR315234, ERR702304–ERR702307, ERR715499–ERR715501, ERR702308, ERR715502–ERR715504, ERR715506–ERR715513, ERR702309, ERR715476, ERR715514–ERR715515, ERR715477, ERR715516–ERR715519, ERR715521, ERR715523–ERR715524, ERR715478–ERR715487, ERR715530–ERR715540, ERR1081365–ERR1081371, ERR1081373–ERR1081376, ERR1081378–ERR1081388, ERR1081372, ERR3634107–ERR3634110, ERR3634112–ERR3634115.

## Abbreviations

BCF: Binary variant call format
CR: Cryptic relatedness
FST: Fixation Index
GRM: Genomic relationship matrix
GS: Genomic selection
GWAS: Genome-Wide Association Studies
IBS: Identity-by-state
IBD: Identity-by-descent
LD: Linkage disequilibrium
MDS: Multidimensional scaling
PCA: Principal components analysis
PCs: Principal components
PS: Population structure
VCF: Variant call format
