# Supplementary material for: PSReliP: an integrated pipeline for analysis and visualization of population structure and relatedness based on genome-wide genetic variant data
Source: BMC Bioinformatics. 2023 Apr 5;24:135. doi: 10.1186/s12859-023-05169-4 (PMC10074814; doi:10.1186/s12859-023-05169-4)
Supplement: Supplementary file 1 — Additional file 1. Notes 1-6. Detailed information about the datasets used, all stages of data analysis and visualisation, their implementation and the user interface. [file 12859_2023_5169_MOESM1_ESM.docx]

Supplementary Information

Table of contents:

Supplementary Notes

1. All steps of the data analysis and visualization of our pipeline and their implementation
2. Additional user interface details described for each tab
3. Commands and parameters used to prepare genetic variant data
4. Details of selecting BioSample accessions and reference genomes, downloading nucleotide sequence data, and preparing genetic variant data
5. Calculating the percentage of variance explained by each principal component
6. Details of datasets with large sample size, results of PSReliP runs and association tests applied to them

Supplementary Figures

Supplementary Tables

Additional References

Supplementary Notes

Supplementary Note 1 All steps of the data analysis and visualization of our pipeline and their implementation

- Analysis stage

The analysis stage, which includes the pre-analysis step, is performed by two bash shell scripts that contained PLINK command lines, Linux bash commands and invoked in-house Perl programs. These bash shell scripts are executed from the command line on Linux-based operating systems and take several arguments from the configuration file. The configuration file is located in the PSReliP installation directory and contains information about the paths to the PLINK executables (1.9 and 2.0), pipeline installation directory, working directory, input files, and parameter values used in the analysis and visualization processes (see Supplementary Table 1 for details). Users must edit the configuration file before executing the bash shell scripts. The details of the setting parameters are described in the configuration file.

PLINK (1.9 and 2.0) is the main software used in all the analysis steps in PSReliP. We used PLINK 2.0 in all cases; however, there are certain commands, such as --ibc, --cluster, --mds-plot, and --distance, that have not yet been implemented in PLINK 2.0; in such a case, we used version 1.9 of the PLINK software, and if these commands are implemented, we will switch the corresponding steps of the analysis to use PLINK 2.0. In the pre-analysis step of PSReliP, the VCF or BCF files are converted into PLINK format files. This step is performed by running the first shell script that takes VCF (possibly gzipped) and BCF files as inputs, which can be either uncompressed or BGZF-compressed (supported by htslib). The main outputs of this step are PLINK 2 binary files in the following formats: PGEN, binary genotype file format; PSAM, format in which sample information is stored; and PVAR, format in which variant information is stored. The newly created PLINK 2 binary files are used as inputs for the following analysis steps. In addition, when this first shell script is run, an allele count report is created and written in PLINK .acount file (produced by –freq with ‘counts’ modifier). This file is used in the analysis stage when loading with --read-freq during the GRM calculation (--make-rel) and PCA run (--pca). Only one filter, such as ‘--max-alleles 2’, is applied in this pre-analysis processing step. It is sufficient to run the first shell script only once for a given set of genetic variants for one specified working directory to prepare the input files for the following analysis. When changing the working directory, it is necessary to start the analysis stage from the beginning and run the first shell script again.

The analysis stage is performed by running the second shell script, which executes all the analysis steps carried out by this pipeline. As mentioned above, during the analysis stage, the following processes are performed: 1) QC and filtering of samples and variants; 2) calculation of basic sample statistics; 3) analysis of PS using PCA, MDS, and clustering; 4) calculation of Wright's FST; and 5) calculation of the IBS, GRM, and KING kinship coefficient matrices. All analyses are carried out using PLINK 1.9 and 2.0 software. While running the second shell script, the PLINK and Linux bash commands are executed sequentially, and many of these commands take input from the previous command and produce an output for the next command. Users can alter multiple parameters used in the analysis steps by appropriately changing their values in the configuration file before running the shell script. Users can run the second shell script multiple times on the given genetic variant dataset using different parameter values and perform the analysis that best matches their data.

PLINK command lines with flags and parameters used

Note that ($) denotes variables with values defined in the configuration file (Supplementary Table 1) and corresponding shell script.

In the PSRelIP pipeline, in all PLINK command lines, the --memory $MAX_MEM_USAGE flag is used.

First shell script:

The PLINK command lines to convert VCF/BCF to PLINK format (PLINK 2 binary fileset will be created):

plink2 --vcf $VCF_FILE_NAME --allow-extra-chr --max-alleles 2 --make-pgen --out binary_fileset

plink2 --bcf $VCF_FILE_NAME --allow-extra-chr --max-alleles 2 --make-pgen –out binary_fileset

The PLINK command line to generate an allele count report, which is a valid input for --read-freq:

plink2 --pfile binary_fileset --allow-extra-chr --freq counts --out plink2.acount

In addition to the PLINK commands, an in-house Perl program was used to create identifiers for all the variants in the binary_fileset.pvar file.

Second shell script:

The PLINK command lines for input filtering:

of samples:

--keep samples.list

--mind $MIND_VAL

of variants:

--chr $RANGE_OF_CHROMOSOMES

--snps-only

--geno $GENO_VAL

--maf $MAF_VAL

The PLINK command lines for basic statistics calculation:

of original dataset and filtered and LD pruned dataset:

--sample-counts

--missing sample-only

of filtered and LD pruned dataset:

--het 'cols=+het,+het'

--ibc

The PLINK command lines for LD-based variant pruning (a pruned subset of variants will be written to plink2.prune.in, which is a valid input for --extract):

window size in kilobase:

--indep-pairwise $LD_WINDOW_SIZE $LD_WINDOW_SIZE_UNITS 1 $LD_THRESHOLD

window size in variant count:

--indep-pairwise $LD_WINDOW_SIZE $LD_STEP_SIZE $LD_THRESHOLD

for excluding all unlisted variants from the current analysis:

--extract plink2.prune.in

The PLINK command line for clustering calculations and multidimensional scaling (MDS) report generation (PLINK 1.9)

--cluster --K $GROUPS_NO --mds-plot 10

The PLINK command line for top 10 principal components (PCs) extraction:

--read-freq plink2.acount --pca

with the 'meanimpute' modifier to request mean-imputes missing genotype calls:

--read-freq plink2.acount --pca meanimpute

The PLINK command line for FST (Pairwise fixation index) estimation between pairs of subpopulations is defined as a categorical phenotype:

--fst CATEGORY --pheno groups.list

with the 'report-variants' modifier to request per-variant FST estimates:

--fst CATEGORY 'report-variants' --pheno groups.list

The PLINK command line for the IBS (identity-by-state) matrix calculation (PLINK 1.9):

--distance square ibs

The PLINK command line for relationship matrix computation:

--read-freq plink2.acount --make-rel square

with the 'meanimpute' modifier to request mean-imputes missing genotype calls:

--read-freq plink2.acount --make-rel meanimpute square

The PLINK command line for KING kinship coefficients computation:

--make-king square

In addition to the PLINK commands, in-house Perl programs were used to reorder samples and their corresponding values in matrices of various types, to edit various values (such as replacing negative values with 0) for visualization purposes, pipelining, and other purposes.

- Visualization stage

To visualize the results of the analysis, we created a web-based visualization stage for PSReliP. We implemented this stage using Shiny technology (https://shiny.rstudio.com/), which provides a dynamic and interactive UI, and developed the Shiny application, an interactive R-based web application. We used the Shiny package in combination with Plotly's R graphing library (https://plotly.com/r/), which allows the creation of interactive graphs and provides basic interactivity, such as zooming in and out, panning graphs, point value display, etc. The all required R packages with their versions are listed in the Availability and Requirements section of the main text of this paper and in the README file of the PSReliP repository on GitHub.

At the end of the PSReliP analysis stage, the second shell script creates a directory with the user-specified name in the configuration file and copies the Shiny application (app.R) into this directory. The results of the analysis and the file containing the arguments for the Shiny app are copied to the ‘data’ subdirectory. Finally, this directory is compressed into a single zip file with the same name as the directory. Typically, a Shiny application can be run locally on RStudio Desktop or deployed in two main ways: on a Linux Shiny Server or on shinyapps.io, which is RStudio's hosting service for Shiny applications (https://shiny.rstudio.com/tutorial/written-tutorial/lesson7/). Accordingly, the resulting zip file can be downloaded for use on another computer or placed in the cloud, or it can be used on the same Linux-based machine to run on Shiny Server or RStudio Desktop after unzipping in the appropriate directory. Running the Shiny application creates interactive data tables, plots, and charts and displays them in a web browser that supports Shiny, such as Google Chrome, Mozilla Firefox, Safari, Microsoft Edge, and Internet Explorer.

R packages used and their functions

The Plotly's R graphing library, which allows the creation of interactive graphics, is also capable of creating a figure that includes different types of subplots. Using the Plotly R library for basic charts, we created grouped and stacked bar charts and line plots as well as a combination of these for basic sample statistics, including GCTA inbreeding coefficient report and scatter plot for the results of PS analysis (PCA plot). In the scatter plot for PCA (bubble chart), marker sizes are variable and marker colors are mapped to a categorical variable. Using Plotly in conjunction with the ‘manhattanly’ R package (https://cran.r-project.org/web/packages/manhattanly/), Manhattan plots for Wright's FST analysis results are created. In Manhattan plots, the genetic variants are plotted with per-variant FST values against their genomic positions. Manhattan plots implemented with the ‘manhattanly’ package have the advantage of adding extra annotation information to each point in these plots. Heatmaps of IBS distances, genetic relationships, and kinship coefficients across all individuals (samples) are created using Plotly in conjunction with the ‘heatmaply’ R package (https://cran. r-project. org/web/packages/heatmaply/). Interactive heatmaps can zoom into a region of interest and allow the checking of values by hovering the mouse over a cell. To visualize the basic statistics of the samples, in addition to charts, tables are created with the ‘DT’ (DataTables) R package (https://cran.r-project.org/web/packages/DT/), which allows users to display their data as tables in the HTML pages and provides filtering, sorting, searching, and other features in the tables. The HTML pages can be saved as standalone HTML files with the necessary JavaScript and CSS embeddings.

Supplementary Note 2 Additional user interface details described for each tab

Details relating to the entire user interface (Figs. 3 and 4):

The parameters used in the analysis stage, which were specified in the configuration file, are shown at the top of Fig. 3 (indicated by ①). The number of samples and variants loaded as well as the number of remaining samples and variants after filtering and linkage disequilibrium (LD) pruning, which were calculated at the analysis stage, are shown at the top of Fig. 3, just below the selected parameters, and are indicated by ②. Fig. 3 ③ shows the download button for PLINK 1.9 .bim file, which is an extended variant information file containing information about all variants used in the analysis.

As can be seen from the two screenshots, the number of samples in the original dataset (143 in Fig. 3) decreased to 141 (Fig. 4) after filtering by the missing genotype rates maximum per-sample (--mind with a value of 0.2). The number of variants also decreased after filtering by maximum missing genotype rates per-variant, minor allele frequency, and LD-based pruning.

Details of the ‘Basic statistics’ tab (Figs. 3 and 4):

Our pipeline performs two types of the basic statistics analysis for both datasets: Sample variant-counts and sample-based missing data counts (Fig.3 ⑥ and Fig 4 ③) calculated by using “--sample-counts” and “--missing sample-only” options, respectively. As shown in Fig. 3, “Sample-based missing data reports" was selected (Fig. 3 ⑥ʹ), and the corresponding report calculated for the original dataset was displayed.

When the LD-based pruning flag was set in the configuration file, two additional analyses are performed. The analyses are as follows: 1) observed and expected homozygous/heterozygous genotype counts for each sample calculated by using “--het ‘cols=+het,+het’” option and 2) three inbreeding coefficients for each sample calculated by using “--ibc” option. As shown in Fig. 4, ‘GCTA inbreeding coefficient report’ was selected (Fig. 4 ③ʹ), and the corresponding report calculated for the filtered and LD-pruned datasets was displayed.

The ‘Basic statistics’ tab offers two types of data representation: charts and tables (Fig. 3 ⑦ and Fig. 4 ④). The ‘Table’ representation (Fig. 3 ⑦ʹ) of the missing data is shown in Fig. 3, and the ‘Chart’ representation (Fig. 4 ④ʹ) (multiple subplots) of the missing data (bar chart) and three inbreeding coefficients (scatter plots with lines) for each sample is shown in Fig. 4.

The original PLINK result files can be downloaded as ZIP files (Fig. 3 ⑧). This ZIP file contains four files: .scount (sample variant-count report), .smiss (sample-based missing data report), .het (method-of-moments F coefficient estimates), and .ibc (GCTA inbreeding coefficient report).

Details of the ‘Population Stratification analysis’ tab (Figs. 5 and 6):

For Population Stratification analysis, we prepared three methods: PCA, normalized PCs (each eigenvector is multiplied by the square root of its eigenvalue), and MDS (Fig. 5 ②). In the example shown in Fig. 5, PCA is selected (Fig. 5 ②ʹ). In PLINK 2.0, by default, the top 10 PCs are extracted from the variance-standardized relationship matrix, and all of these components are used in the visualization stage of our pipeline.

Fig. 6a shows a similar pattern, as shown in Fig. 5. The TEJ group (Fig. 6a ①) and indica varieties, which included the IND and AUS groups (Fig. 6a ③), were separated from each other by PC1, while the TRJ group (Fig. 6a ②) was separated from the others by the PC2. Fig. 6b shows the PCA plot for the same data (Runs E in Supplementary Table 3); however, the marker colors in Fig. 6b indicate the clusters calculated using PLINK. The clustering function or user-specified groups can be set as a parameter in the configuration file. In addition, users can specify the number of clusters that are biologically interesting or easily interpretable. As shown in Fig. 6b, the TEJ and IND groups are divided into two separate clusters along the first and second PCs, respectively, which coincides with the PCA analysis.

As with other types of analysis, on the ‘Population Stratification analysis’ tab, we prepared a button to download analysis result files (Fig. 5 ⑦), which were are either PLINK output files such as .eigenvec (PCs), .eigenval (eigenvalues), and .mds or generated by our pipeline as a normalized_plink_pca.txt file, which contained normalized PCs calculated using PLINK output files.

Details of the ‘Wright's FST estimation’ tab (Fig. 7):

The Manhattan plot of FST values for variants does not load in the browser if large number of genetic variants are included in the analysis (after filtering and pruning). Therefore, we plot chromosomes/contigs one at a time or the entire genome region only if the number of variants is ≥ 100 and ≤ 100,000. Users can switch these views by changing the corresponding values from the drop-down list (Figs. 7 ③ and S5 ②). According to the selected values of this list, Fig. 7 shows the distribution of FST on the Manhattan plot for all chromosomes, while Fig. S5 shows the Manhattan plot for only chromosome 9. To reduce the loading time of the Manhattan plot containing a large amount of data, we added a drop-down list with a range of FST values (0–0.9) (Figs. 7 ④ and S5 ③). Accordingly, only those variants with FST values ≥ 0.1 were displayed in the plot shown in Fig. S5.

The download button (Fig. 7 ⑥) allows the user to download files obtained with the PLINK --fst command and containing FST estimates between the two selected subpopulations. It can be a single file, that is, fst.summary (all-population-pairs Wright's FST report) or two files, .fst.summary and .fst.var (per-variant Wright's FST report for one population pair), depending on the number of groups/clusters (per-variant FST estimates are calculated if the number of groups/clusters is ≤ 5). The user can find the original files in the ‘data’ subdirectory of the Shiny app directory.

Details of the ‘IBS and GRM calculation & Kinship Coefficients estimation’ tab (Fig. 8):

Due to the long loading time of the heatmaps if the number of samples is large and that the information in these heatmaps is difficult to see without strong zooming, we create heatmaps only if the number of samples is ≤ 400. If the number of samples is > 400, heatmaps are not created, but users retain the ability to download the original results of this type of analysis.

The results of IBS, GRM, and Kinship coefficient calculations can be downloaded by clicking the ‘Save data as a zip file’ button (Fig. 8 ⑥). The files are as follows: .mibs (identity-by-state matrix), .rel (relationship matrix), .king (KING-robust kinship coefficient matrix), and corresponding .id (Sample ID list) files.

Supplementary Note 3 Commands and parameters used to prepare genetic variant data

Analysis tools

Java (JDK 1.8.0_111)

BWA (bwa-0.7.17)

SamTools (v1.12)

GATK (v4.2.0.0)

Trimmomatic (v0.39)

Picard (v2.25.5)

Creating index of the genome

samtools faidx genome.fa

java -jar picard.jar CreateSequenceDictionary REFERENCE= genome.fa OUTPUT= genome.dict

bwa index genome.fa

Preprocessing of Illumina paired-end reads (per sample)

TRIMMOMATIC_OPT=”ILLUMINACLIP:adapters.fa:2:30:10 LEADING:20 TRAILING:20 SLIDINGWINDOW:4:20 MINLEN:36”

java -jar trimmomatic-0.39.jar PE -phred33 read.r1.fastq.gz read.r2.fastq.gz read.pe.r1.fastq.gz read.se.r1.fastq.gz read.pe.r2.fastq.gz read.se.r2.fastq.gz $TRIMMOMATIC_OPT

Alignment of Illumina reads to the reference genome (per sample)

$ bwa mem -M genome.fa read.pe.r1.fastq.gz read.pe.r2.fastq.gz | samtools sort -o pe_masterPsrt.bam -

$ samtools view -u -q 20 pe_masterPsrt.bam | samtools sort -n -T pe -O bam - | samtools fixmate -O bam - pe_fixmate.bam

$ samtools view -u -f 0x2 pe_fixmate.bam | samtools sort -T pe -O bam -o pe_pSrt.bam -

$ samtools index pe_pSrt.bam

Removal of PCR duplicates (per sample)

$ java -jar picard.jar MarkDuplicates INPUT=pe_pSrt.bam OUTPUT=pe_rmdup.bam METRICS_FILE=mark_duplicates.metrics MAX_RECORDS_IN_RAM=1000000 REMOVE_DUPLICATES=true

$ samtools index pe_rmdup.bam

Variant detection by GATK HaplotypeCaller (per sample)

$ gatk HaplotypeCaller --input pe_rmdup.bam --output sample_n.g.vcf.gz --reference genome.fa --emit-ref-confidence GVCF

Combining per-sample files into a multi-sample VCF file by GATK CombineGVCFs

IN_GVCFs='--variant sample_1.g.vcf.gz --variant sample_2.g.vcf.gz --variant sample_n.g.vcf.gz …’

$ gatk CombineGVCFs -R genome.fa -O multi_sample.g.vcf.gz $IN_GVCFs

Genotyping and filtering variants by GATK

$ gatk GenotypeGVCFs --include-non-variant-sites -R genome.fa -V multi_sample.g.vcf.gz -O multi_sample.genotyped.vcf.gz

VAR_FILTER_OPT='QD < 2.0 || FS > 60.0 || MQ < 40.0 || MQRankSum < -12.5 || ReadPosRankSum < -8.0'

$ gatk VariantFiltration --reference genome.fa --variant multi_sample.genotyped.vcf.gz --output multi_sample.filtered.vcf.gz --filter-expression "${VAR_FILTER_OPT}" --filter-name "FILTER"

gatk SelectVariants --reference genome.fa --variant multi_sample.filtered.vcf.gz --output multi_sample.varonly.vcf.gz --exclude-filtered --select-type-to-include SNP --select-type-to-include INDEL

Supplementary Note 4 Details on selecting BioSample accessions and reference genomes, downloading nucleotide sequence data and preparing genetic variant data

To divide samples into groups and obtain SRA accessions of each sample, we downloaded the metadata from the NCBI BioSample database (https://www.ncbi.nlm.nih.gov/biosample/) using the BioSample accessions selected as described in the ‘Preparing data for case studies’ subsection of the main text of this paper and listed them in Supplementary Tables 4 and 5. Using the obtained SRA Run accessions (Supplementary Tables 4 and 5), we downloaded nucleotide sequence data as fastq files from the European Nucleotide Archive (ENA) (https://www.ebi.ac.uk/ena/browser/home) and used them to generate genetic variant data. The chromosome-level genome assemblies, such as IRGSP-1.0 (RefSeq assembly accession: GCF_001433935.1; BioProject: PRJDB1747) (Kawahara et al. [38]) and M_zebra_UMD2a (RefSeq assembly accession: GCF_000238955.4; BioProject: PRJNA60369) (Conte and Kocher [39]), were used as reference genomes for variant calling for rice and Malawi cichlids data, respectively. For reference genome selection for the analysis of Malawi cichlids, our method differs from the method described by Malinsky et al. [37], in which Illumina short reads were aligned to the M. zebra reference assembly version 1.1 (MetZeb1.1_prescreen) (Brawand et al. [45]), which is a scaffold-level genome assembly. There was also a difference in the set of accessions used in the analysis and in the programs used to perform the PCA analysis and genome-wide FST calculations, which was the PLINK software in our pipeline and the smartpca program from the eigensoft v5.0.2 software package. However, the PCs calculated using the PLINK –pca command were very similar to the PCs calculated by eigensoft's smartpca program, and both programs used the same Hudson method to estimate FST values. Despite the described differences, the results obtained by us for the assessment of PS, which are described in the ‘Results obtained in case studies’ subsection of the main text of this paper, are comparable to those published in the article by Malinsky et al. [37].

Supplementary Note 5 Calculating the percentage of variance explained by each principal component

The proportion of variance explained by each principal component (PC) was calculated by dividing the PC’s corresponding eigenvalue by the sum of all eigenvalues.

The PLINK --pca command extracts the top principal components from the variance-standardized relationship matrix computed by --make-rel/--make-grm (https://www.cog-genomics.org/plink/2.0/strat#pca).

To calculate the proportion of variance explained by each principal component, we relied on the following reference sources.

Butler [46] described as follows:

Let A be an n×n matrix.

• Matrix A has n eigenvalues (including each according to its multiplicity).

• The sum of n eigenvalues of A is the same as the trace of A (i.e., the sum of the diagonal elements of A).

Parellada [47] described as follows:

1. PCA relies on the singular value decomposition of the covariance matrix (Cov(Data)).
2. The trace is the sum of variance values in the diagonal of Cov(data).
3. The trace is the sum of eigenvalues.

Thus, it follows that the sum of the eigenvalues is the same as the sum of the diagonal in the covariance matrix. We calculated the principal components from the variance-standardized relationship matrix computed by --make-rel (to calculate a straight covariance matrix, ‘cov’ modifier and --make-rel ‘cov’ command should be used); additionally, we calculated the percentage of the variance explained by each principal component as follows:

Percentage of the variance explained by n-th eigenvector (n-th PC) = (n-th eigenvalue / the sum of diagonal entries of the relationship matrix) * 100

Supplementary Note 6 Details of datasets with large sample size, results of PSReliP runs and association tests applied to them

To demonstrate that our pipeline is applicable to a dataset with a large sample size, we used data published in scientific papers (Mural et al. [48] and Lin et al. [49]) and applied our pipeline to these datasets. The VCF genotype file we used as input for our pipeline was created in the study by Mural et al. [48], contains data on genome-wide genetic variants of maize varieties and is available for public download [50]. From this VCF file, we extracted two datasets consisting of 1,049 and 380 samples and analyzed them with our pipeline. The dataset of 1,049 samples contains all the samples in SupplementalDataFileS2_GenotypesAndTraitValues.xlsx (Supplemental Data for Ref. 48) and we used them together with their IDs (column ‘GenotypeID’) and the group names (column ‘ConsensusSubPopulationOrHeterotic Group’) into which they were divided. The dataset of 380 samples contains samples for which the values of the ‘LeafCuticularConductance6_H’ column in the same Excel file do not equal ‘NA’. As shown in SupplementalDataFileS3_PhenotypeMetaData.xlsx (Supplemental Data for Ref. 48), the column ‘LeafCuticularConductance6_H’ contains phenotype values that were collected in the study by Lin et al. 2020 (column ‘SourcePaper’) in 2017 (column ‘FieldExperimentYears’) in Maricopa_AZ (column ‘FieldExperimentLocations’). From the study by Lin et al. [49], we used only this set of phenotype values, which is one of the four sets collected in this study for the ‘maize leaf cuticular conductance’ that differ by year and location of the experiment.

Both datasets were analyzed by our pipeline and the results are shown in Figs. S15-S18. Fig. S15 shows the results of the same run of PSReliP, in which (a) shows multiple subplots for the ‘Method-of-moments F coefficient estimates’ report; (b) shows a Manhattan plot of FST values for a chromosome 1 that were calculated between cluster 0 and cluster 1 using complete-linkage hierarchical clustering; (c) shows a 2-component PCA plot for the PC1 and PC2 in which the colors of the data points correspond to 5 clusters. Figs. S15-S16 and S17a show the results of the population structure analyses that are very similar to those reported by Mural et al. [48] and Lin et al. [49], respectively. Figs. S17b and S18a-b show the results of IBS, GRM, and Kinship coefficient calculation.

The 380 samples dataset, along with the results of running our pipeline applied to it, was also used in the genetic association tests carried out by Tassel 5 and Emmax for the ‘maize leaf cuticular conductance’ phenotype. We conducted the genetic association tests to show an example of how the results of the detection of population structure and cryptic relatedness between individuals performed by our pipeline can be used in downstream analysis. We show the results of these tests in Fig. S19. As can be seen from this figure, the results of the tests carried out with different tools and using different data to indicate genetic relatedness vary in terms of p-values but are similar in those regions of the genome where peaks are observed.

Supplementary Figures


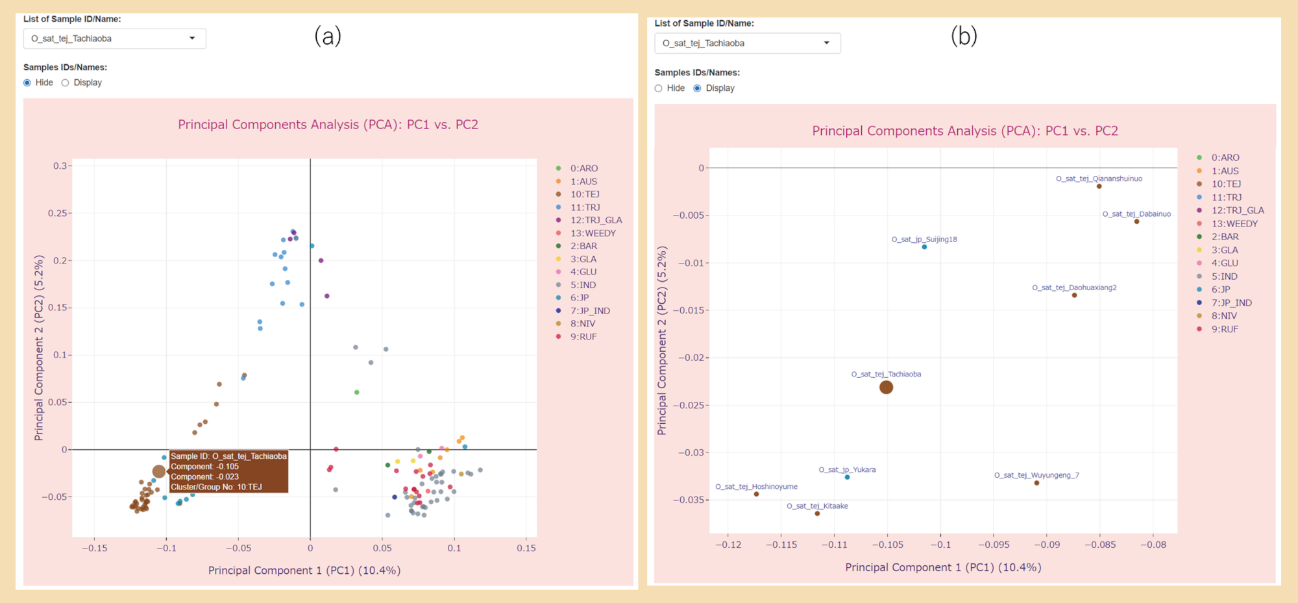


Fig. S1 An illustration of the main features of the PCA plots created in PSReIP

(a, b) The interactive 2-component PCA plot displays the first and second principal components (PC1/PC2) for 141 accessions of rice varieties, with data point colors corresponding to 14 rice type groups. The parameters used are shown in Supplementary Table 3 (Run A). (a) The sample selected from the drop-down list titled ‘List of Sample ID/Name’ is displayed is a larger font size. Hovering the mouse pointer over the marker (sample) in the scatter plot will display annotation information related to that sample. (b) IDs or names of the samples are displayed by checking the ‘Display’ value in the radio button titled ‘Samples IDs/Names’. In Fig. S1b, we have zoomed in on the bottom left corner of the plot shown in Fig. S1a and illustrated the Plotly's zoom functionality. Users can zoom in and out of Plotly graphs by clicking the ‘Zoom in’ button (+ icon) and ‘Zoom out’ button (- icon) on the Modebar.


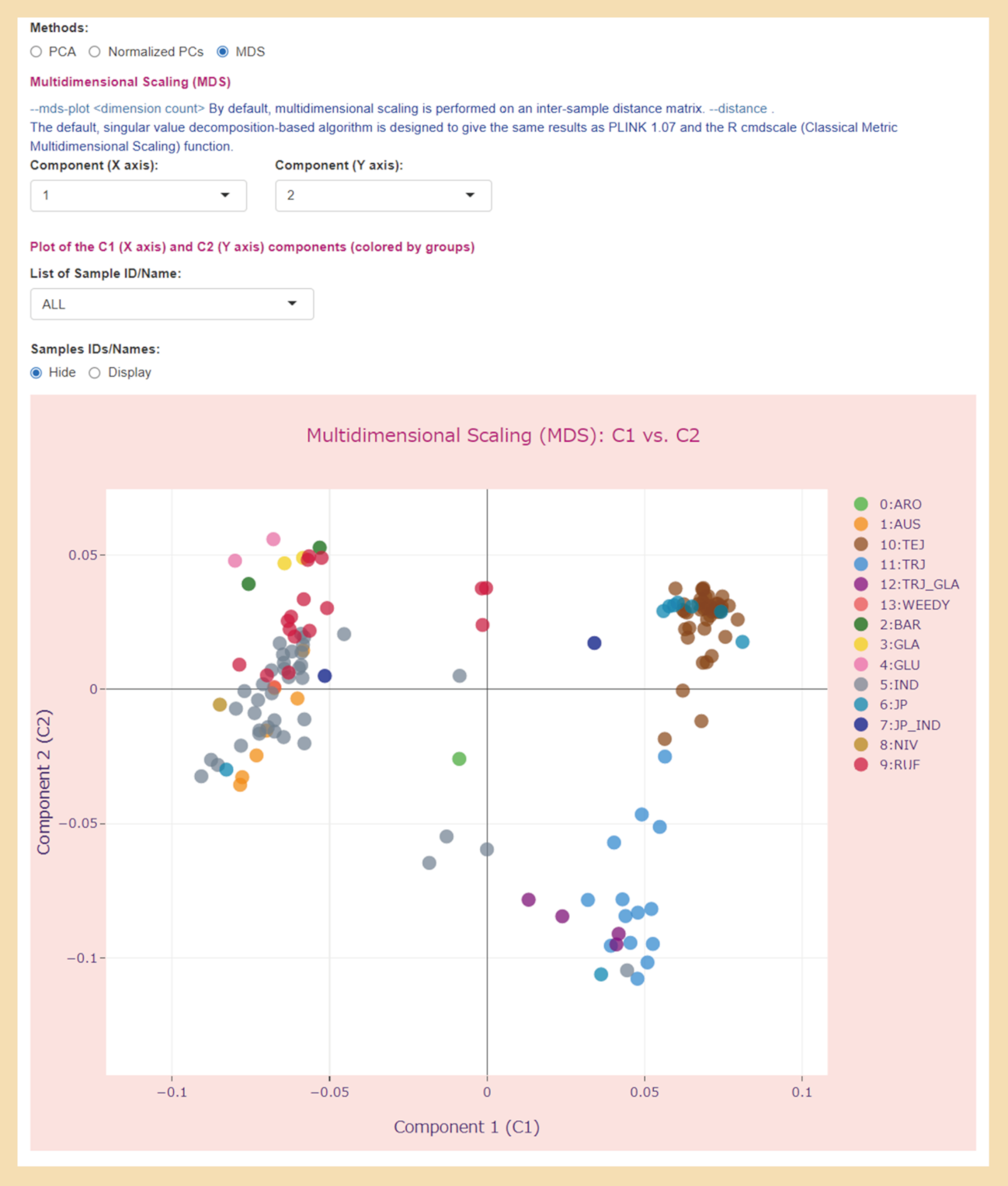


Fig. S2 User interface for results of Population Stratification analysis with the two-dimensional MDS plot

The parameters used are listed in Supplementary Table 3 (Run A). The interactive two-dimensional MDS plot displays the first (Component 1, C1) and second dimension of MDS (Component 2, C2) for 141 accessions of rice varieties, with data point colors corresponding to 14 rice type groups. A PCA plot of the first two principal components of the same dataset is shown in Fig. 5.


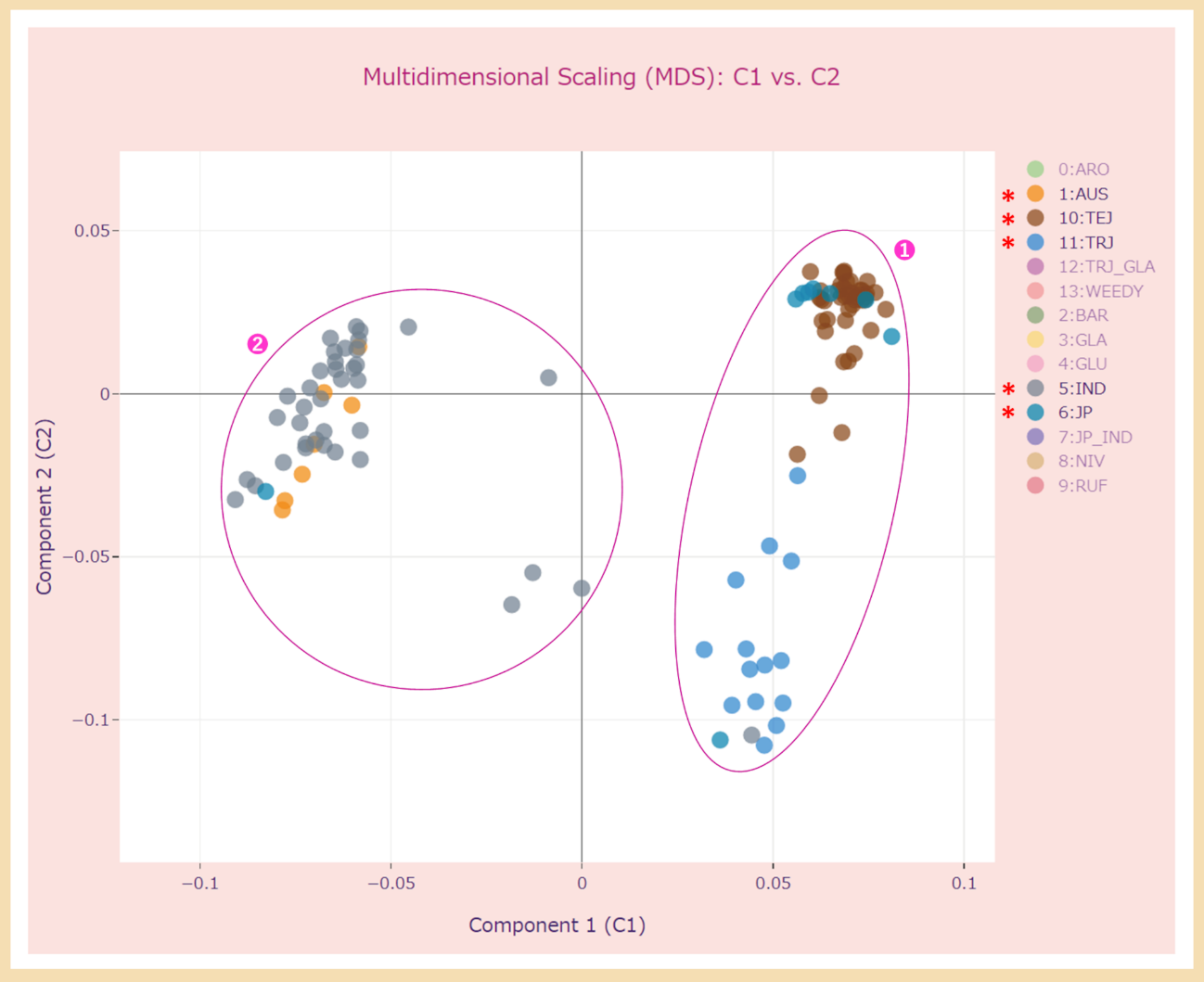


Fig. S3 An illustration of Plotly's ability to toggle the visibility of the data series. Plotly legends are interactive

Trace parts of the same legend group can be hidden or shown at the same time by clicking the corresponding legend item. This two-dimensional MDS plot shows samples from only five groups, JP, TEJ, and TRJ (indicated by ①) and IND with AUS (indicated by ②), since only these groups are selected in the legend (in this figure, we marked them by an asterisk). The parameters used are listed in Supplementary Table 3 (Run A).


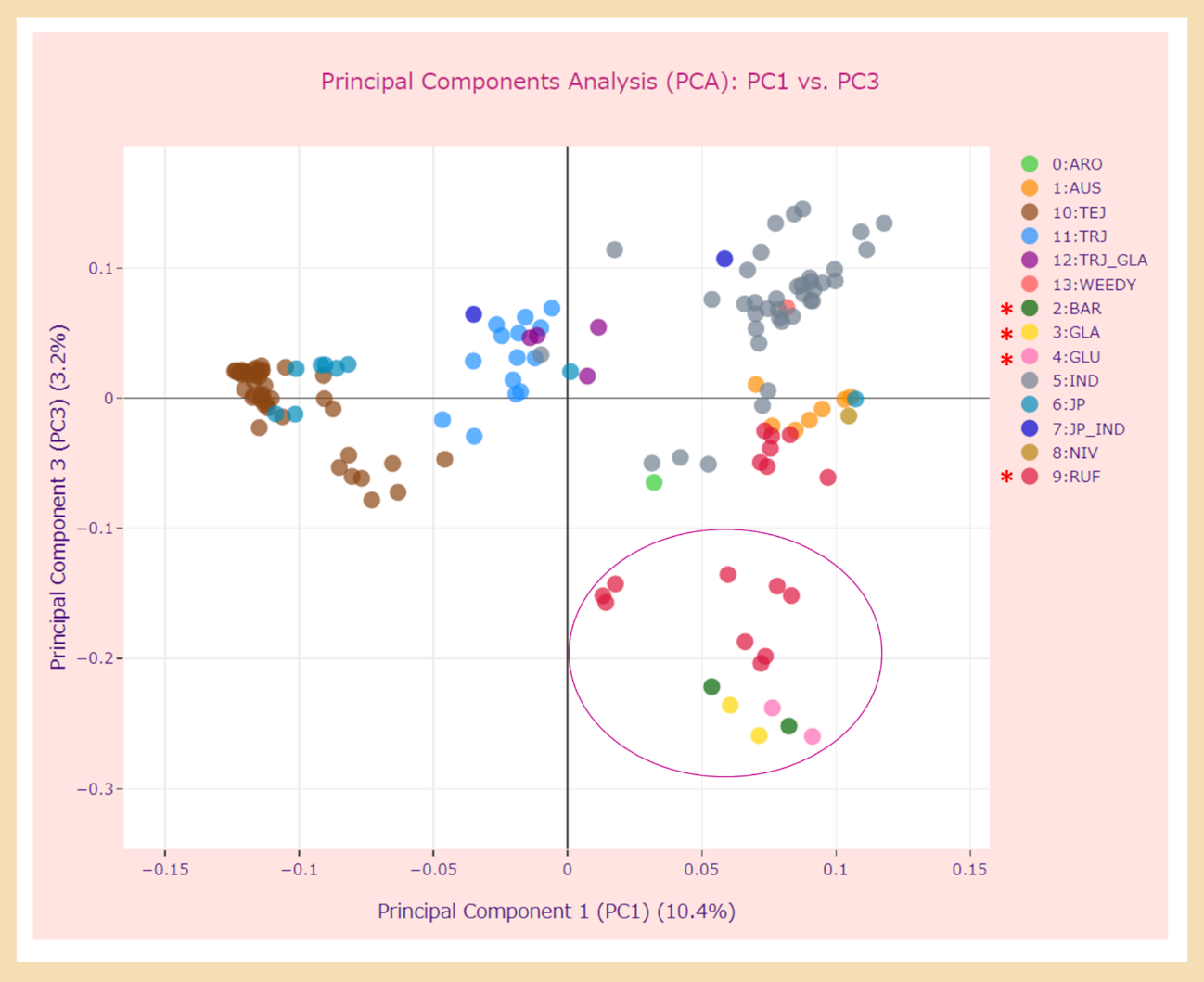


Fig. S4 The interactive 2-component PCA plot of the first and third principal components (PC1/PC3)

The parameters used are listed in Supplementary Table 3 (Run A). PC1 explained 10.4% of the variance, while PC3 explained 3.2%. Samples from groups such as BAR (Oryza barthii), GLA (Oryza glaberrima), and GLU (Oryza glumaepatula) as well as some samples from the RUF (Oryza rufipogon) group, which are indicated in the figure by an oval, were separated along the third principal component from samples from other groups, including the groups of Oryza sativa. A PCA plot of the first two principal components of the same dataset is shown in Fig. 5.


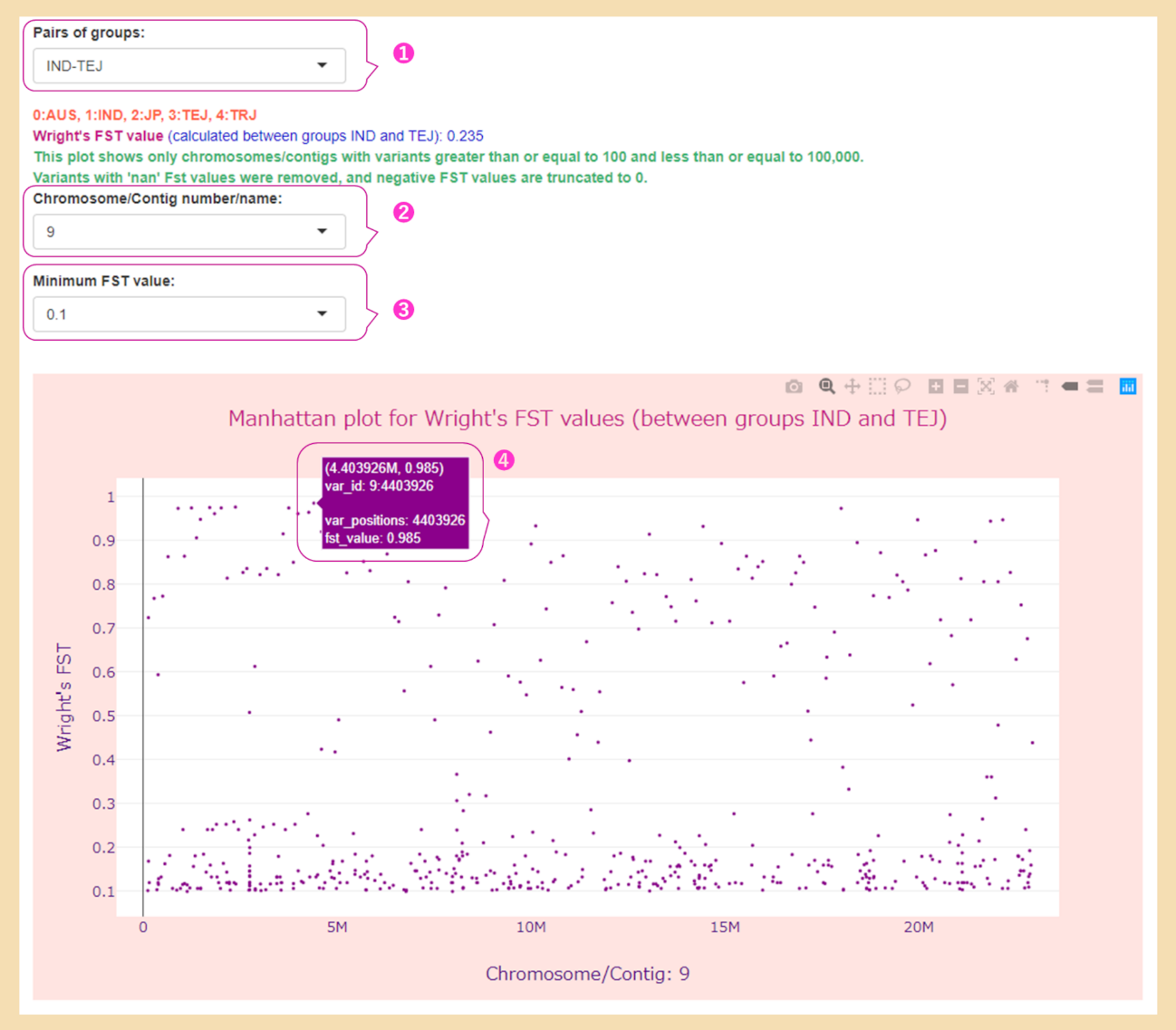


Fig. S5 User interface for FST estimation with the Manhattan plot of FST values for a single chromosome

The parameters used are listed in Supplementary Table 3 (Run C). ① The drop-down list with values of all pairs of subpopulations (groups or clusters of samples) for which the FST values are shown on the Manhattan plot, with the selected value ‘IND-TEJ’; ② the drop-down list with the value ‘ALL’ or the name of the chromosome/contig (in this case, chromosome 9), which contains variants whose number is ≥100 and ≤100,000; ③ the drop-down list with a range of FST values (0–0.9) to filter the data displayed in the Manhattan plot, with a selected value of 0.1; ④ annotation information related to the variant, such as identifier, position on the chromosome, and FST value, which will be displayed on hovering the mouse over the variant. The interactive Manhattan plot shows Wright's FST values for variants on chromosome 9 calculated between the IND and TEJ rice groups.


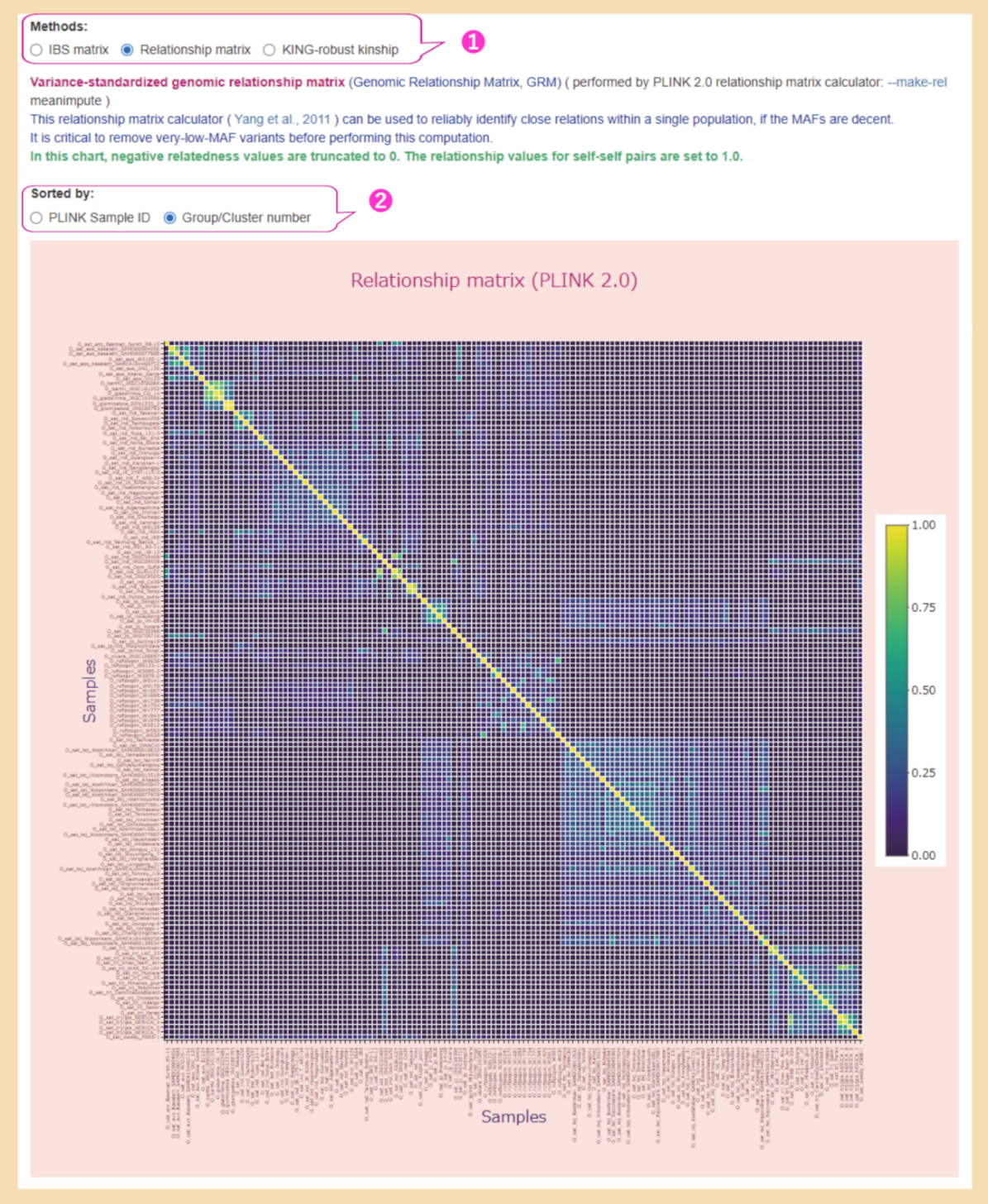


Fig. S6 User interface for results of IBS, GRM, and Kinship coefficient calculation with the heatmap of GRM

The parameters used are listed in Supplementary Table 3 (Run A). ① A radio button for choosing any of the three methods for this type of analysis, of which the ‘Relationship matrix’ value is selected; ② a radio button to select the sort order of the samples for the heatmap with the value ’Group/Cluster number’ selected, meaning that the order is according to the groups/clusters to which the samples are assigned. The interactive heatmap represents the Relationship matrix for 141 accessions of rice varieties, in which the samples are ordered by ‘Group/Cluster number’.


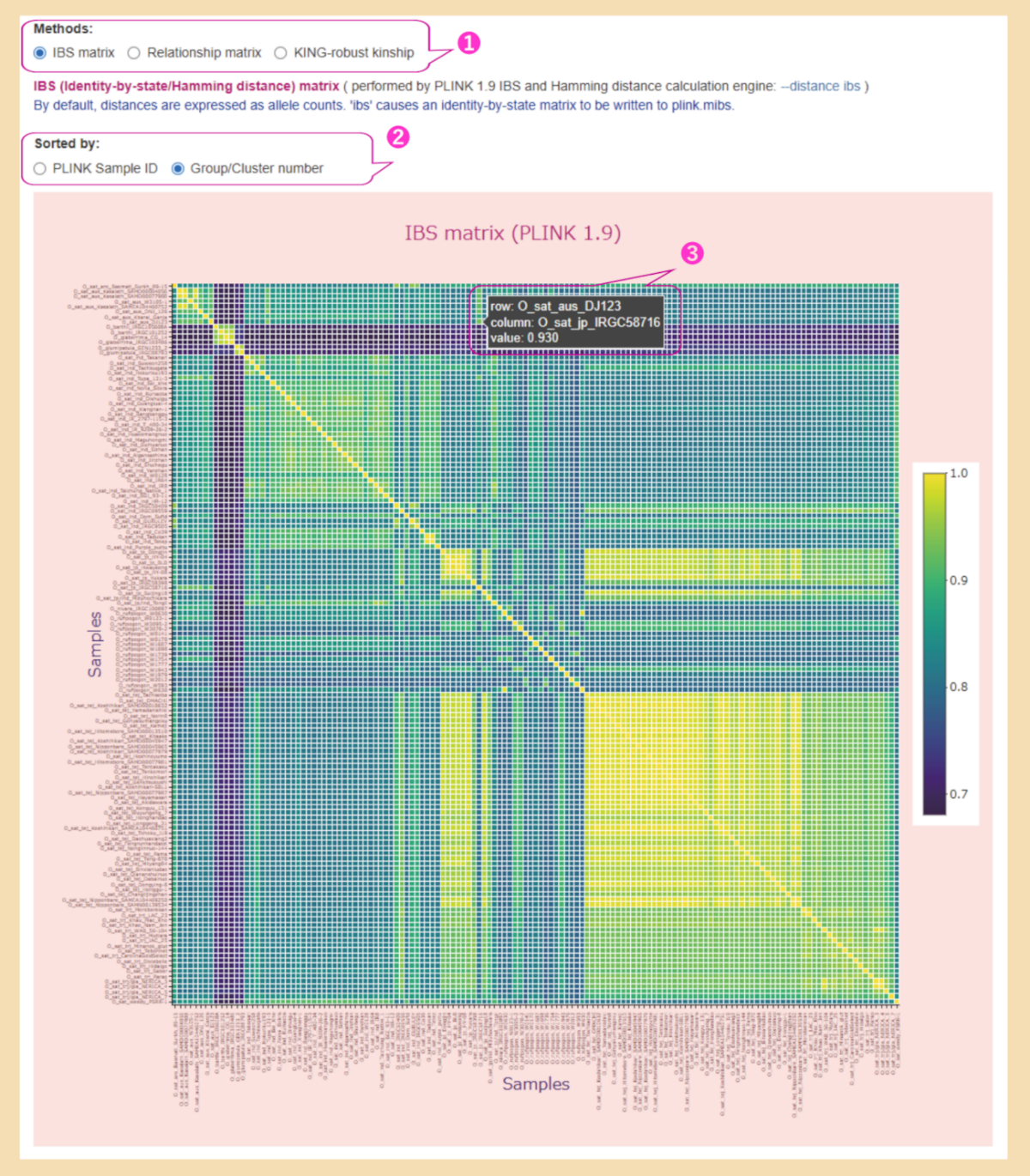


Fig. S7 User interface with the heatmap of IBS calculated for the dataset not pruned for LD

The parameters used are listed in Supplementary Table 3 (Run B). ① A radio button for choosing any of the three methods for this type of analysis, of which the ‘IBS matrix’ value is selected; ② a radio button to select the sort order of the samples for the heatmap with the value ’Group/Cluster number’ selected; ③ the IBS value for the pair of samples and their ID/name displayed by hovering the mouse over the colored square. The interactive heatmap represents the IBS matrix for 141 accessions of rice varieties, in which the samples are ordered by ‘Group/Cluster number’. Compared with the heatmap of IBS shown in Fig. 8, in which the IBS matrix was calculated for the dataset pruned for LD, the values of the IBS matrix in this map are higher, as in the example of the same pair of samples, indicated by ⑤ in Fig. 8 (IBS value is 0.859) and ③ in Fig. S7 (IBS = 0.930), respectively.


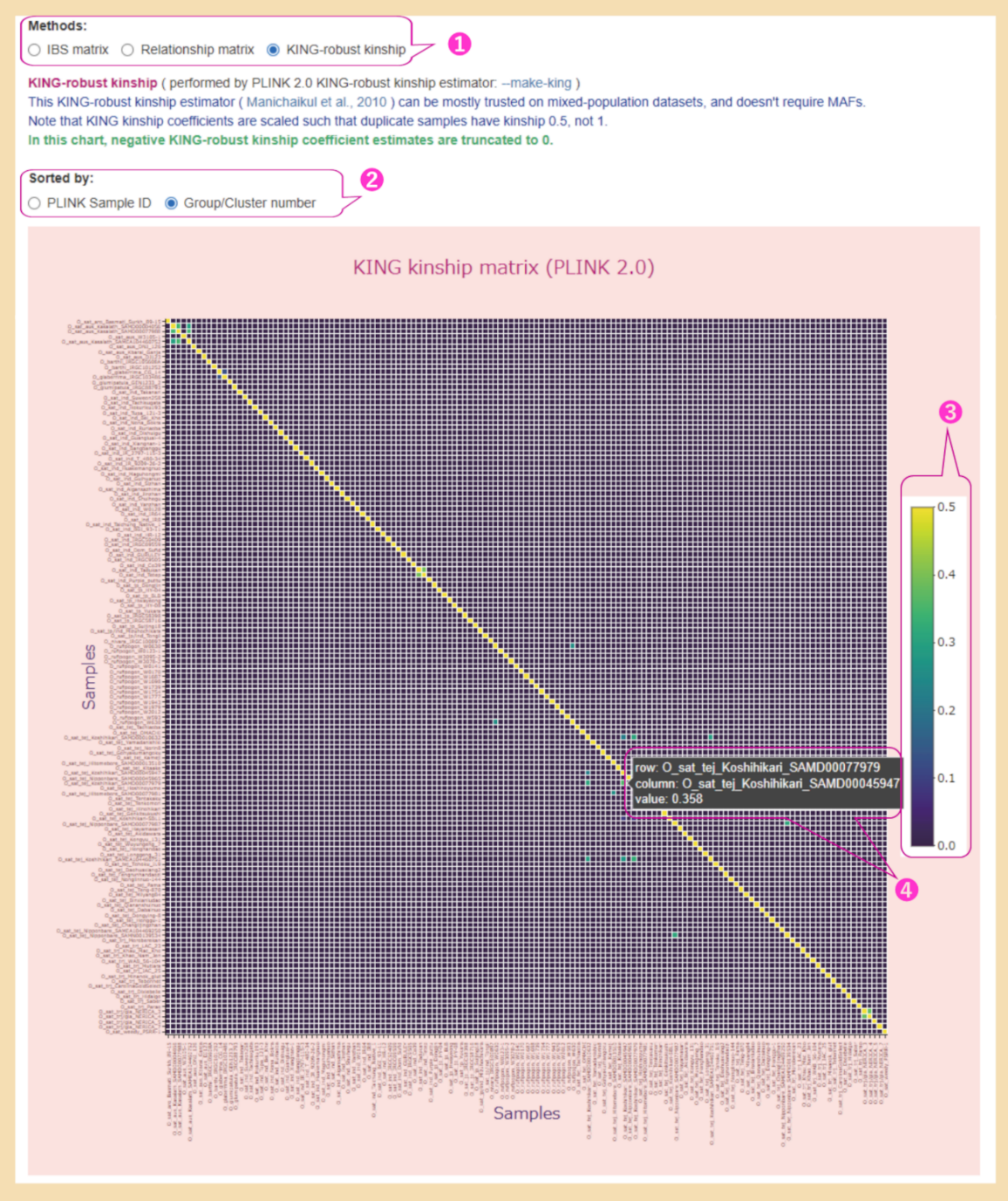


Fig. S8 User interface with the heatmap of KING-robust kinship coefficients

KING-robust kinship coefficients were estimated for data that were not pruned for LD, and the --maf value was set to 0.01 (Run B in Supplementary Table 3). ① A radio button for choosing any of the three methods used for this type of analysis, of which the ‘KING-robust kinship’ value is selected; ② a radio button to select the sort order of the samples for the heatmap with the value ’Group/Cluster number’ selected; ③ the gradient color bar mapping colors to their corresponding values (note that the duplicate samples have a kinship 0.5, not 1); ④ the KING-robust kinship coefficient for pairs of samples and their IDs/names displayed by hovering the mouse over the colored square. The interactive heatmap represents the KING-robust kinship matrix for 141 accessions of rice varieties, in which the samples are ordered by ‘Group/Cluster number’.


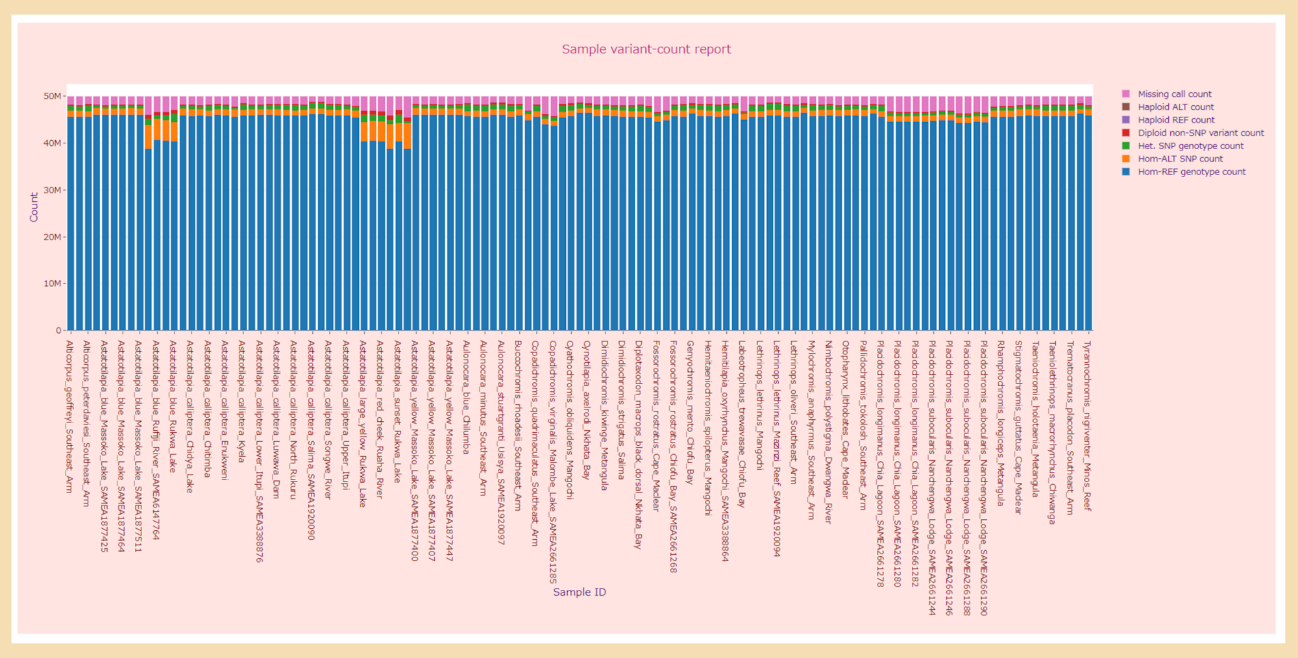


Fig. S9 The interactive stacked bar chart for the ‘Sample variant-count report’ from the ‘Basic statistics’ tab

The interactive stacked bar chart displays the ‘Sample variant-count report’ for the original dataset of 119 accessions of Malawi cichlids, reporting the number of observed variants subdivided into various classes. The parameters used are listed in Supplementary Table 3 (Run F). The classes of observed variants are shown in the legend of the stacked bar chart. Most of the observed variants belonged to the ‘Hom-REF genotype’ class when the reads were mapped to the M_zebra_UMD2a reference genome (RefSeq assembly accession: GCF_000238955.4; BioProject: PRJNA60369).


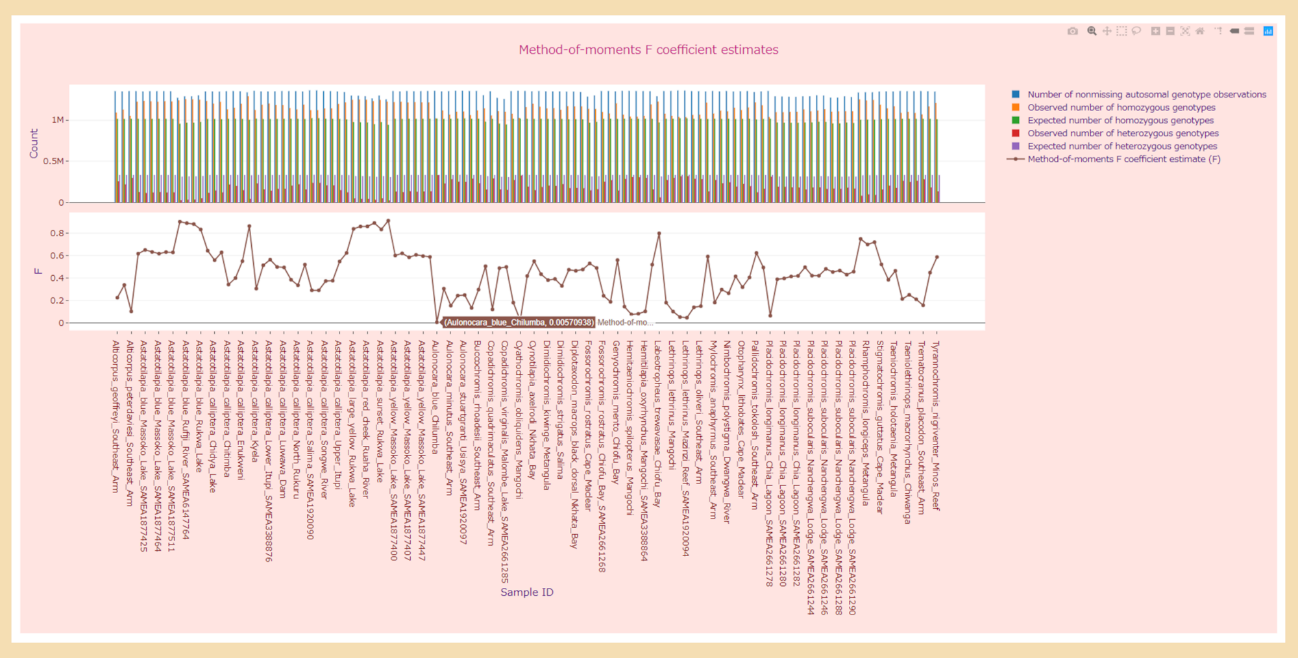


Fig. S10 Multiple subplots for the ‘Method-of-moments F coefficient estimates’ report from the ‘Basic statistics’ tab

Interactive multiple subplots, namely the grouped bar chart and line plot, display the ‘Method-of-moments F coefficient estimates’ report for the ‘After filtering’ dataset of 119 accessions of Malawi cichlids (Run F in Supplementary Table 3). Each column of the report is listed in the legend and is shared across multiple subplots. The low expected heterozygosity seen in this figure indicates high homozygosity and low genetic diversity in Malawi cichlids.


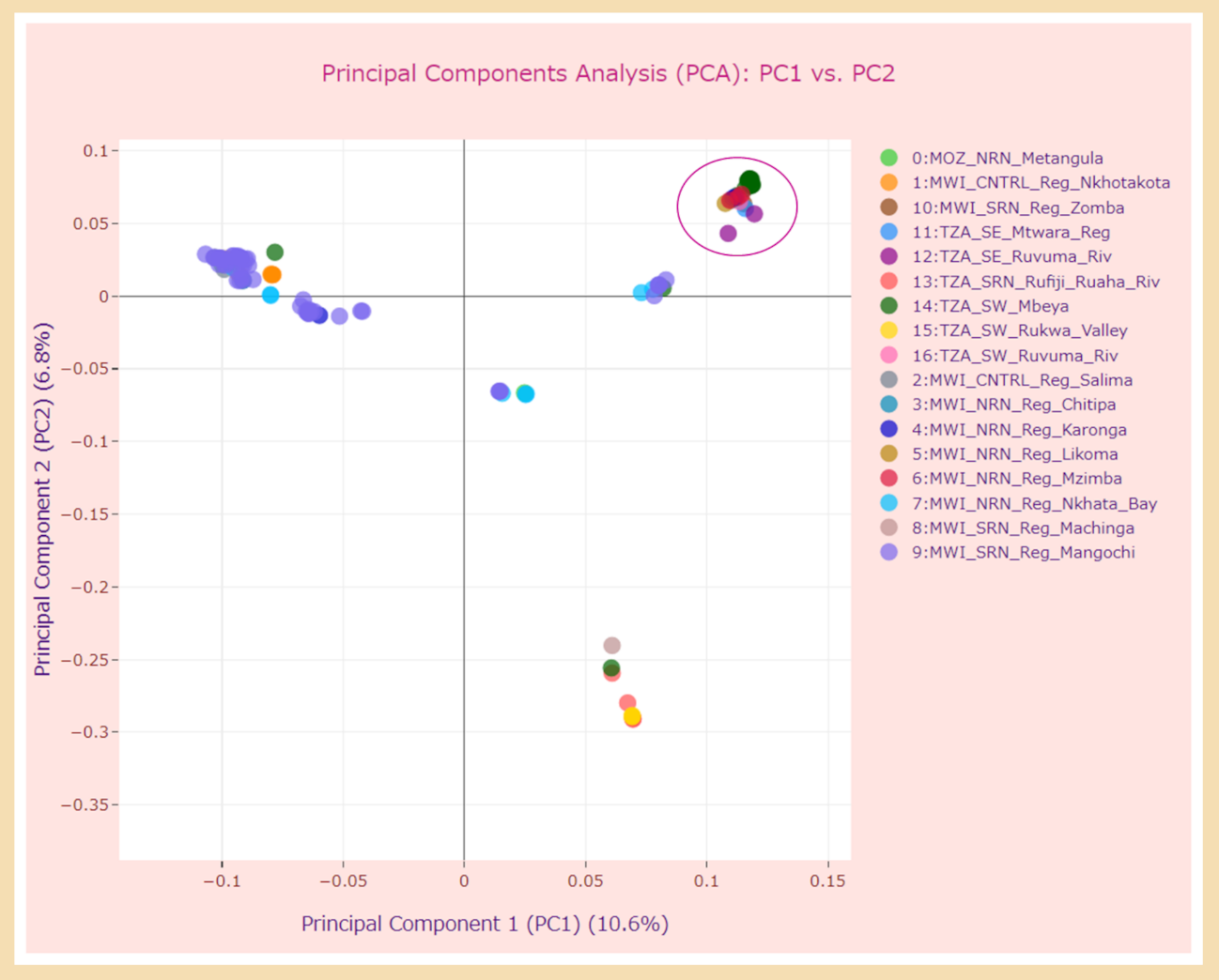


Fig. S11 PCA plot with data points colored according to sampling locations.

The interactive 2-component PCA plot displays the first and second principal components (PC1/PC2) for the 119 accessions of Malawi cichlids. The parameters used are listed in Supplementary Table 3 (Run F). The colors of the data points correspond to the 17 groups (we divided the samples depending on the geographic location of the places where they were taken; see the Supplementary Notes for details). The groups that were separated from each other by the first and second principal components correlated well with the eco-morphological groups. The group of the Astatotilapia calliptera species is indicated in the figure by an oval.


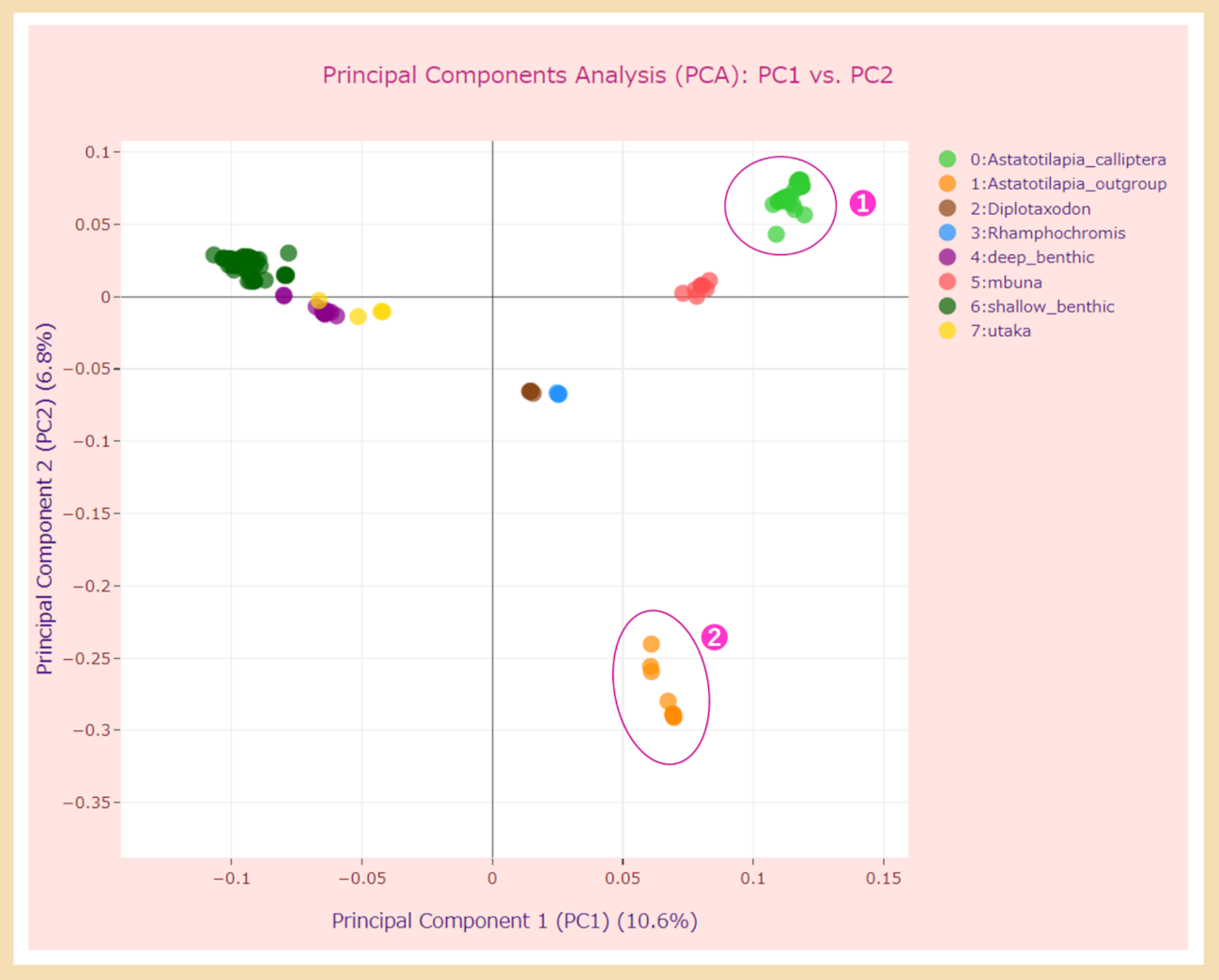


Fig. S12 PCA plot with data points colored according to eco-morphological groups.

The interactive 2-component PCA plot displays the first and second principal components (PC1/PC2) for the 119 accessions of Malawi cichlids. The parameters used are listed in Supplementary Table 3 (Run G). ① The group of samples of Astatotilapia calliptera species; ② the samples from the ‘outgroup Astatotilapia’. The colors of the data points correspond to the seven eco-morphological groups of Malawi cichlids and the ‘outgroup Astatotilapia’, which consists of the individuals of the Astatotilapia calliptera species, but lives in an area outside the Lake Malawi catchment. The groups that were separated from each other by the first and second principal components correlated well with the eco-morphological groups indicated by corresponding colors.


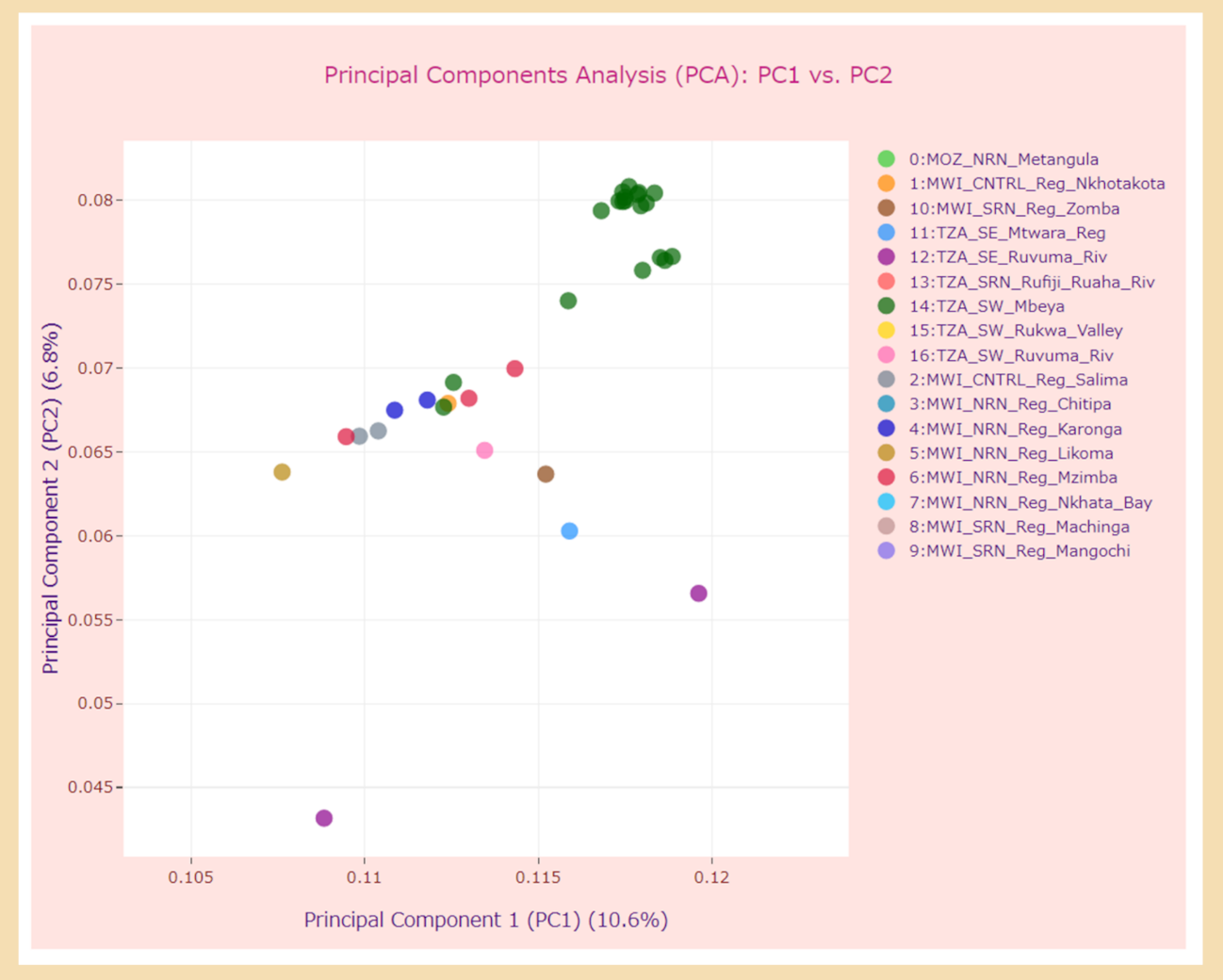


Fig. S13 PCA plot for the Astatotilapia calliptera species with data points colored according to sampling locations

The interactive 2-component PCA plot for the first and second principal components (PC1/PC2) for 119 accessions of Malawi cichlids displayed the same dataset as that in Fig. S11 but is zoomed in on the group of Astatotilapia calliptera species, indicated by an oval in Fig. S11. The parameters used are listed in Supplementary Table 3 (Run F). For some individuals, an association between genetic similarity and sampling locations can be seen in this plot.


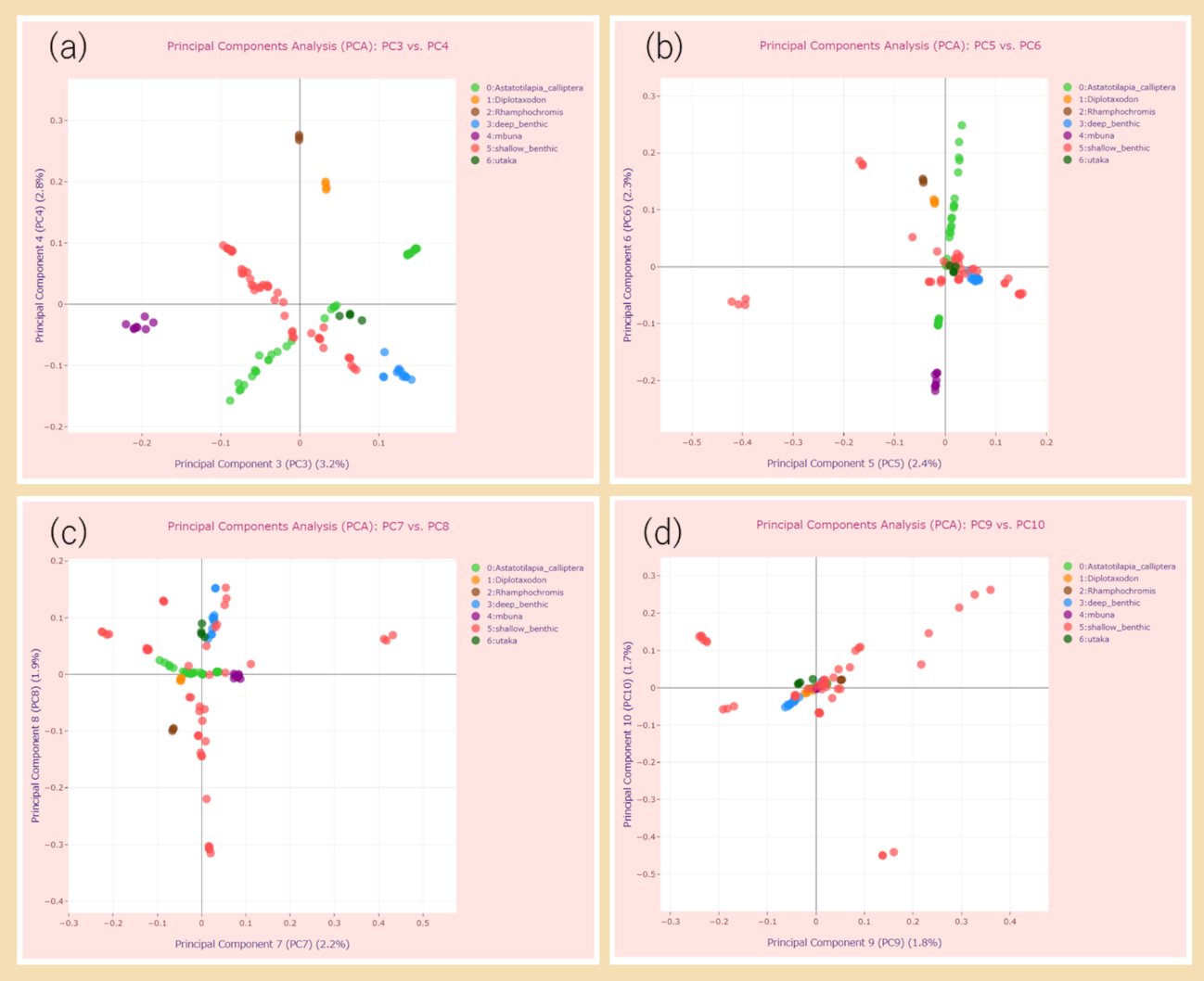


Fig. S14 The 2-component PCA plots of top 3-10 principal components for 109 accessions of Malawi cichlids

The parameters used are listed in Supplementary Table 3 (Run H). The data point colors correspond to seven eco-morphological groups. The 2-component PCA plot for the first and second principal components (PC1/PC2) for the same dataset are shown in Fig. 9.


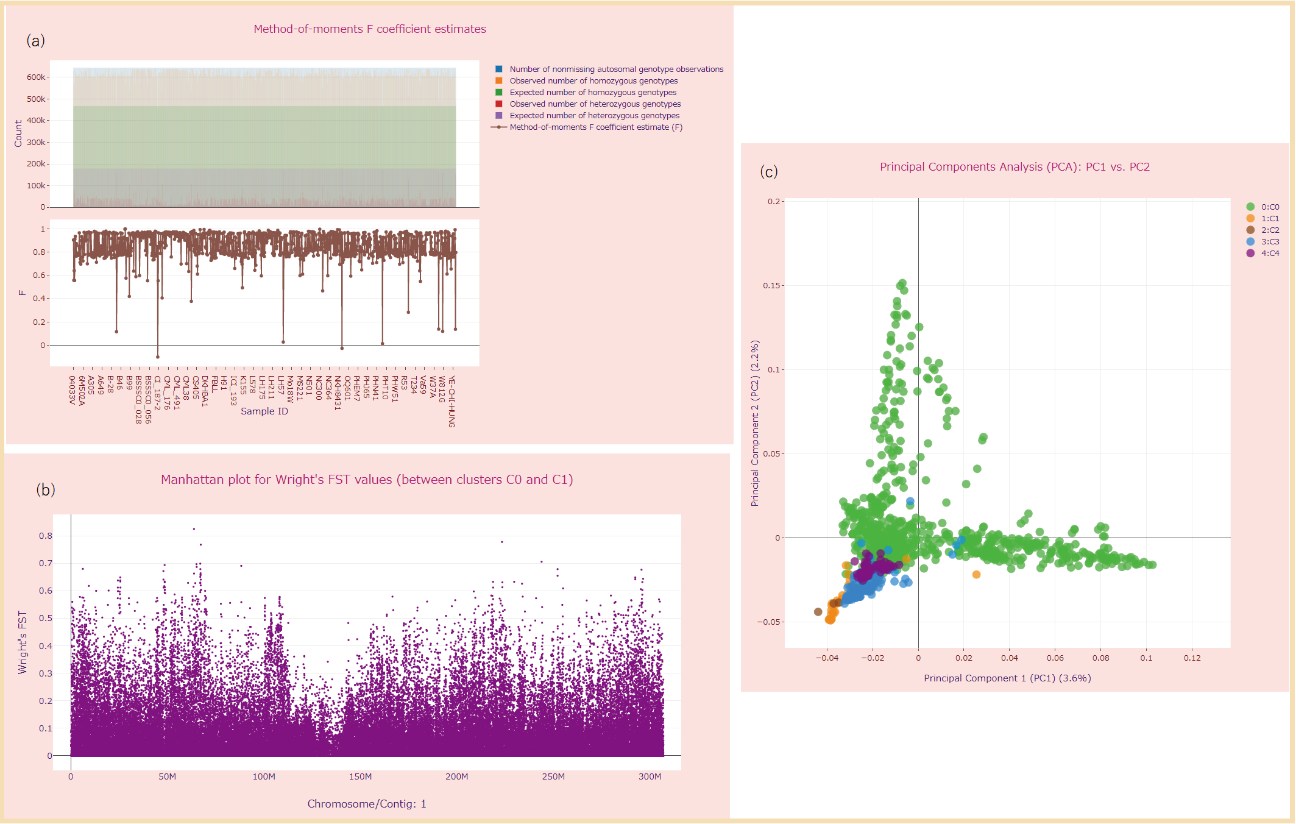


Fig. S15 User interface for the dataset of 1049 accessions of Maize

The VCF file of this dataset was downloaded from the website associated with Ref. [50]. The parameters used in the analysis stage are: geno: 0.2; mind: 0.2; maf: 0.01, LD-based pruning: indep-pairwise 100 5 0.2; r2 threshold: 0.2; minimum final cluster count: 5. (a) Multiple subplots for the ‘Method-of-moments F coefficient estimates’ report from the ‘Basic statistics’ tab. (b) Manhattan plot of FST values for a single chromosome. The FST values between cluster 0 and cluster 1 were calculated using complete-linkage hierarchical clustering performed by PLINK 1.9. (c) The 2-component PCA plot for the first and second principal components (PC1/PC2). The data point colors in this plot correspond to 5 clusters.


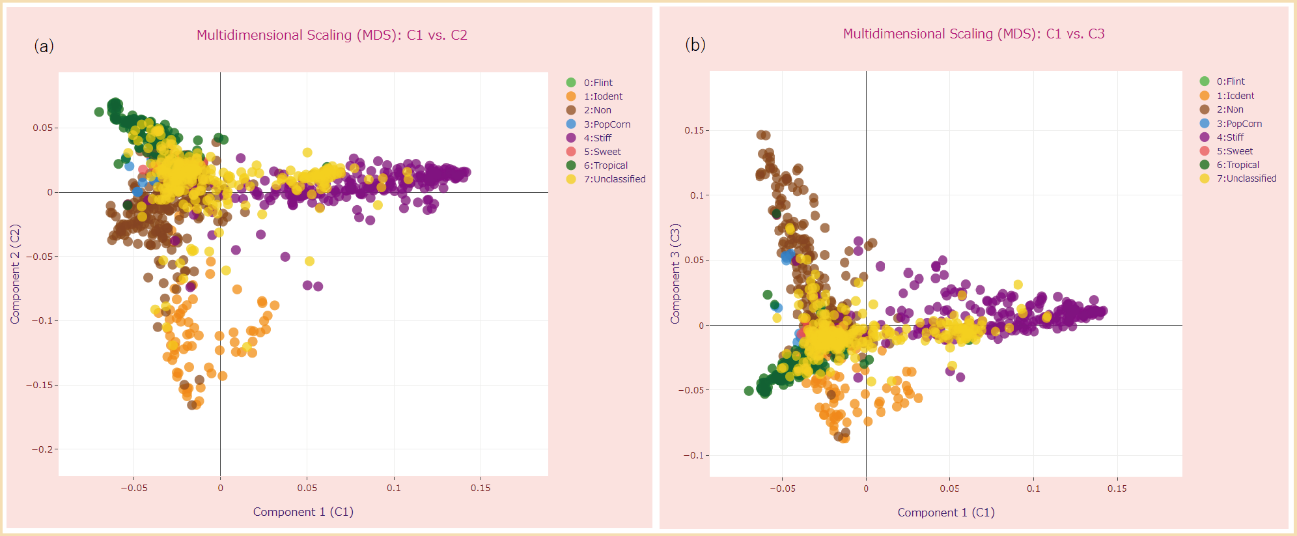


Fig. S16 The interactive two-dimensional MDS plots for 1049 accessions of Maize

The VCF file of this dataset was downloaded from the website associated with Ref. [50]. The parameters used in the analysis stage are: geno: 0.2; mind: 0.2; maf: 0.01, LD-based pruning: indep-pairwise 100 5 0.2; r2 threshold: 0.2; number of groups of samples: 8. (a) The interactive two-dimensional MDS plot displays the first (Component 1, C1) and second dimension of MDS (Component 2, C2). (b) The interactive two-dimensional MDS plot displays the first (Component 1, C1) and third dimension of MDS (Component 3, C3). The data point colors in these plots correspond to the 8 groups of maize types.


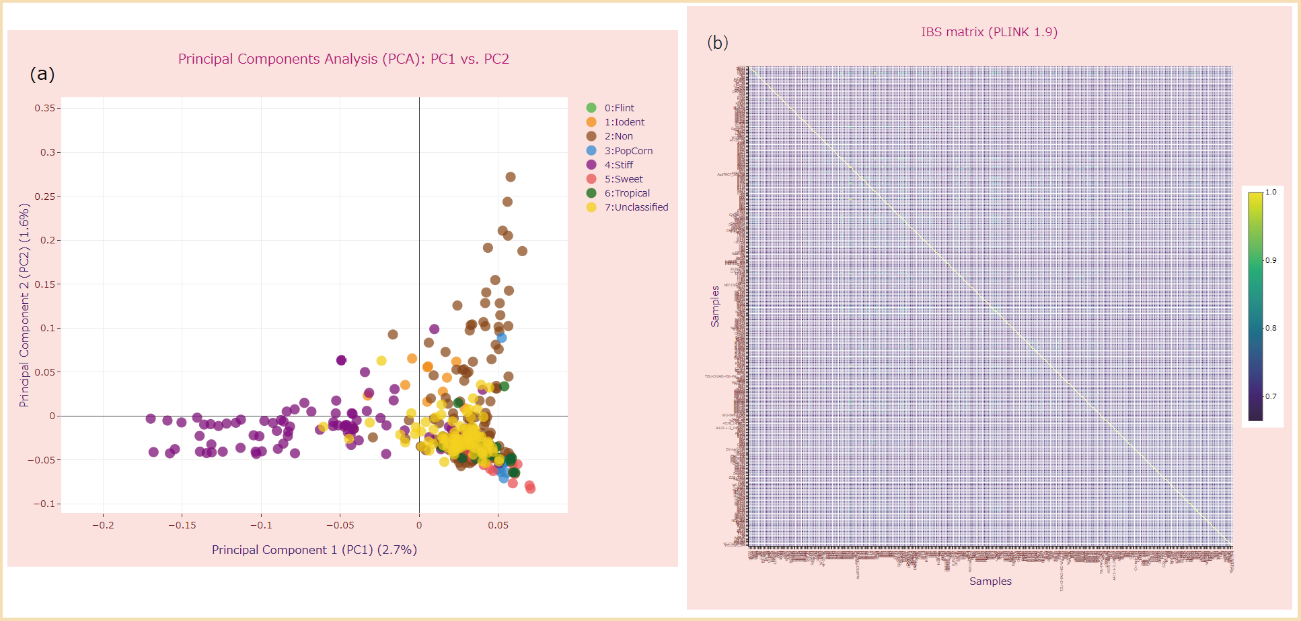


Fig. S17 User interface for the dataset of 380 accessions of Maize

The VCF file of this dataset was downloaded from the website associated with Ref. [50]. The parameters used in the analysis stage are: geno: 0.2; mind: 0.2; maf: 0.05, LD-based pruning: indep-pairwise 100 5 0.2; r2 threshold: 0.2; number of groups of samples: 8. (a) The interactive 2-component PCA plot for the PC1 and PC2. The data point colors in this plot correspond to the 8 groups of maize types. (b) The heatmap of IBS without scaling.


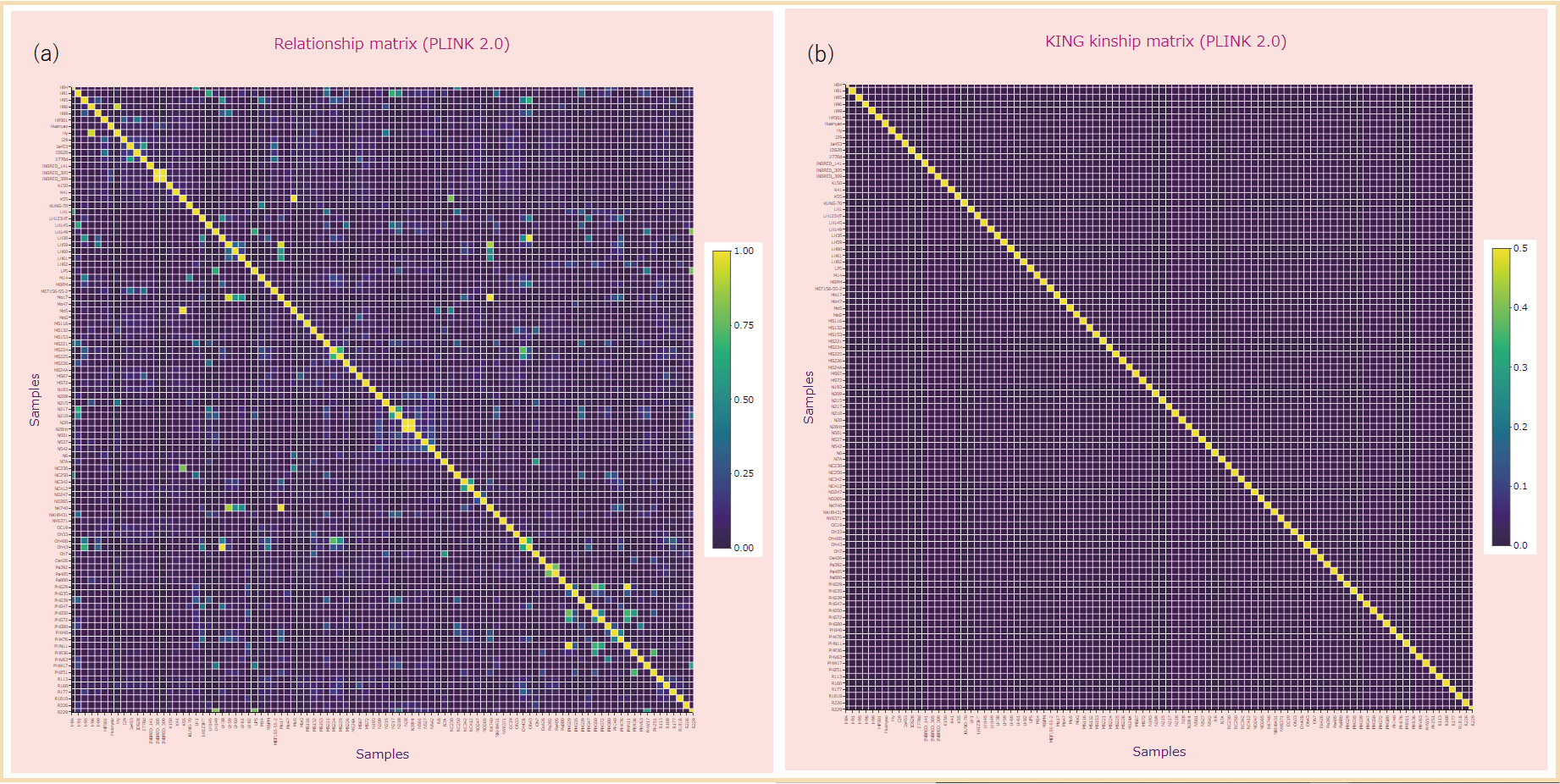


Fig. S18 User interface with the heatmaps of GRM and KING-robust kinship coefficients

The interactive heatmaps display the GRM and KING-robust kinship matrix for the dataset of 380 accessions of Maize. The VCF file of this dataset was downloaded from the website associated with Ref. [50]. (a) The parameters used in the analysis stage are: geno: 0.2; mind: 0.2; maf: 0.05, LD-based pruning: indep-pairwise 100 5 0.2; r2 threshold: 0.2; number of groups of samples: 8. The heatmap of the GRM, zoomed in at top right. (b) The parameters used in the analysis stage are: geno: 0.2; mind: 0.2; maf: 0.01, LD-based pruning: no; number of groups of samples: 8. The heatmap of KING-robust kinship coefficients, zoomed in at top right.


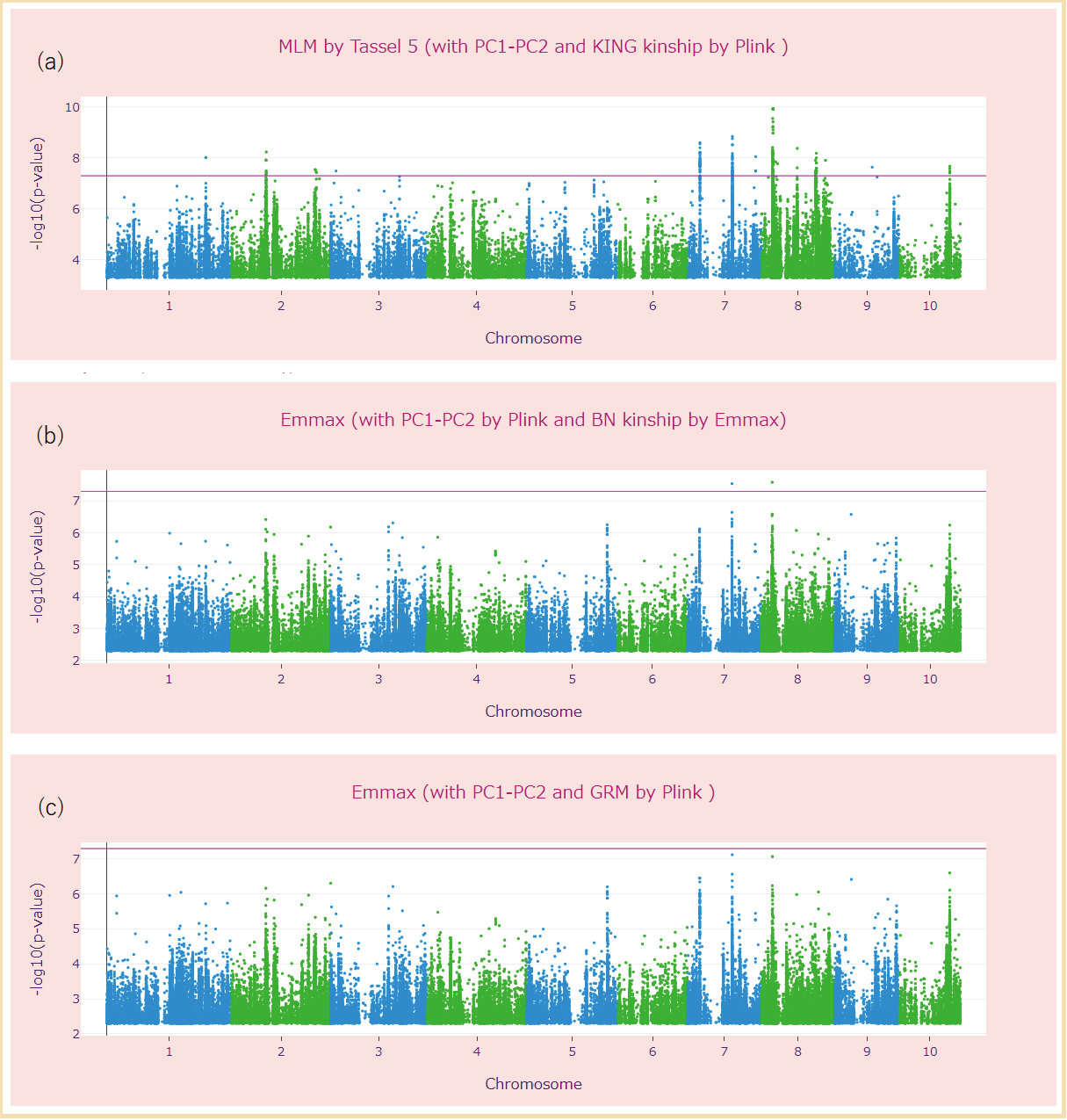


Fig. S19 Manhattan plots of results of the association tests

An example of using the PSRelIP pipeline results for downstream analysis. Association tests results for the ‘maize leaf cuticular conductance’ phenotype collected in Maricopa, AZ in 2017. The dataset used was created by Mural et al. [48] and Lin et al. [49]. The VCF file of this dataset was downloaded from the website associated with Ref. [50]. Genetic association tests were carried out using Tassel 5 and Emmax software. Manhattan plots display genomic coordinates on the X-axis plotted against −log10 of p-values. (a) Results of MLM performed by Tassel 5 with PC1-PC2 (Fig. S17a) and KING-robust kinship coefficients (Fig. S18b) performed by PLINK. (b) Results of the association test performed by Emmax with PC1-PC2 (Fig. S17a) performed by PLINK and BN (Balding-Nichols) matrix performed by Emmax. (c) Results of the association test performed by Emmax with PC1-PC2 (Fig. S17a) and GRM (Fig. S18a) performed by PLINK. The purple horizontal line represents the genome-wide significance threshold of p-value = 5.0×10­8.

Supplementary Tables

Supplementary Table 1 List of parameters used in the pipeline configuration file

Note: ^a^ represents the version of the PLINK executable used in our pipeline; ^b^ denotes a quotation from the PLINK 2.0 User Manual (https://www.cog-genomics.org/plink/2.0/); ^c^ denotes a quotation from the PLINK 2.0 Command-line help; and ^d^ denotes a quotation from the PLINK 1.9 User Manual (https://www.cog-genomics.org/plink/1.9/).

Supplementary Table 2 Parameter values, the number of samples and variants, and the required time (first shell script).

Note: Computing time represents the time it took to complete runs on eight threads and 8000 MB RAM of memory and 32 threads and 32000 MB RAM of memory; Max alleles represents the PLINK --max-alleles flag, which filters out variants with more than a given number of alleles; Number of samples and variants represents the number of samples and variants calculated at the analysis stage. In the PSReliP pipeline, the –threads and --memory flags are used in the PLINK command lines, and the values of these parameters can be specified in the configuration file (see Supplementary Table 1 for details).

Supplementary Table 3 Parameter values, the number of samples and variants, and the required time (second shell script).

Note: Computing time represents the time it took to complete runs on 8 threads and 8000 MB RAM of memory and 32 threads and 32000 MB RAM of memory; Setting parameters represents the parameters specified in the configuration file; Types of variants represents the type of variants, such as “SNPs” or “SNPs and InDels,” included in the analysis (“SNPs” indicates that the PLINK --snps-only flag was used); Geno represents the PLINK --geno flag, which filters out all variants with missing call rates exceeding the provided value; Mind represents the PLINK --mind flag, which filters out all samples with missing call rates exceeding the provided value; Maf represents the PLINK --maf flag, which filters out all variants with allele frequency below the provided threshold; Meanimpute represents the usage of the PLINK 'meanimpute' modifier to request the mean imputation of missing genotype calls (in --pca and --make-rel commands); Clustering represents the usage of the PLINK --cluster command to perform complete-linkage hierarchical clustering; Number of groups represents the number of groups/clusters provided by users or calculated by PLINK; LD-based pruning represents the usage of the PLINK --indep-pairwise command to produce a pruned subset of variants that are in approximate linkage equilibrium with each other (it takes three parameters: window size in variant count (vc) or kilobase (kb), variant count to shift the window (step size), which is required to be 1 when a kilobase window is used, and r2 threshold); Number of samples and variants represents the number of samples and variants calculated at the analysis stage; Filtered variants represents the number of variants remaining after filtering; and Filtered and pruned variants represents the number of variants remaining after filtering and LD-based pruning (if it was used).

Supplementary Table 4 BioSample, BioProject, and SRA accessions of the rice varieties used in the case study

Note: ^a^ represents the BioSample accession numbers from the BioSample database (https://www.ncbi.nlm.nih.gov/biosample/); ^b^ represents the BioProject accession numbers from BioProject (https://www.ncbi.nlm.nih.gov/bioproject/); and ^c^ represents the SRA accession numbers from the Sequence Read Archive (SRA) based on the source database (SRA, European Bioinformatics Institute (EBI), or DNA Data Bank of Japan (DDBJ)).

Supplementary Table 5 BioSample, BioProject, and SRA accessions of Malawi cichlids used in the case study

Note: ^a^ represents the BioSample accession numbers from the BioSample database (https://www.ncbi.nlm.nih.gov/biosample/); ^b^ represents the BioProject accession numbers from BioProject (https://www.ncbi.nlm.nih.gov/bioproject/); and ^c^ represents the SRA accession numbers from the Sequence Read Archive (SRA) based on the source database (European Bioinformatics Institute (EBI)).

Supplementary Table 6 List of rice samples with their names and BioSample accession numbers and groups

Note: The asterisk (*) represents the data used in the PSRelIP pipeline.

Supplementary Table 7 List of Malawi cichlid samples with their names, BioSample accession numbers, and groups.

Note. *Eco-morphological group*: We grouped the samples into seven eco-morphological groups based primarily on Malinsky et al. (2018) [37] and additional information from Duncan and Martin (2019) [51]. Additionally, we added one more group such as the ‘outgroup Astatotilapia’. *Geographic location of the sampling site* and *Group abbreviation (sampling location)*: Using the information on sampling locations recorded in the NCBI BioSample database as the value of the description field, we divided the samples into 17 groups according to the geographic location of the locations where they were taken. We grouped sampling locations by country, geographic regions within those countries, and administrative divisions within them. The asterisk (*) represents the data used in the PSRelIP pipeline.

Additional References:

1. Brawand D, Wagner CE, Li YI, Malinsky M, Keller I, Fan S, et al. The genomic substrate for adaptive radiation in African cichlid fish. Nature. 2014;513(7518):375-81. doi:10.1038/nature13726.
2. Butler D. Facts About Eigenvalues By Dr David Butler. https://www.adelaide.edu.au/mathslearning/ua/media/120/evalue-magic-tricks-handout.pdf. Accessed 14 Mar 2022.
3. Parellada A. Why is the sum of eigenvalues of a PCA equal to the original variance of the data? CrossValidated, StackExchange. https://stats.stackexchange.com/questions/266864/why-is-the-sum-of-eigenvalues-of-a-pca-equal-to-the-original-variance-of-the-dat (2019). Accessed 14 Mar 2022.
4. Mural RV, Sun G, Grzybowski M, Tross MC, Jin H, Smith C, et al. Association mapping across a multitude of traits collected in diverse environments in maize. Gigascience 2022;11:giac080. doi: 10.1093/gigascience/giac080.
5. Lin M, Matschi S, Vasquez SM, Chamness J, Kaczmar N, Baseggio M et al. Genome-Wide Association Study for Maize Leaf Cuticular Conductance Identifies Candidate Genes Involved in the Regulation of Cuticle Development, G3 Genes|Genomes|Genetics 2020;10(5):1671-1683. doi: 10.1534/g3.119.400884.
6. Mural R, Sun G, Grzybowski M, Tross MC, Jin H, Smith C, et al. (2022): Maize_WiDiv_SAM_1051Genotype.vcf.gz genotype file. figshare. Dataset. https://doi.org/10.6084/m9.figshare.19175888.v1. Accessed 05 Dec 2022.
7. Duncan EE, Martin JG. Adaptive diversification of the lateral line system during cichlid fish radiation. iScience. 2019;16:( )1-11. doi:10.1016/j.isci.2019.05.016.
